# Synthesis, Characterization, and Cytotoxicity Studies of N-(4-Methoxybenzyl) Thiosemicarbazone Derivatives and Their Ruthenium(II)-*p*-cymene Complexes

**DOI:** 10.3390/molecules27227976

**Published:** 2022-11-17

**Authors:** Mónica Martínez-Estévez, Soledad García-Fontán, Saray Argibay-Otero, Inmaculada Prieto, Ezequiel M. Vázquez-López

**Affiliations:** 1 Departamento de Química Inorgánica, Campus Universitario, Universidade de Vigo, E-36310 Vigo, Spain; 2 Metallosupramolecular Chemistry Group, Galicia South Health Research Institute (IIS Galicia Sur) SERGAS-UVIGO, E-36213 Vigo, Spain; 3 Departamento de Química Física, Campus Universitario, Universidade de Vigo, E-36310 Vigo, Spain

**Keywords:** ruthenium, metallocene, thiosemicarbazone, cytotoxicity

## Abstract

The reaction of [Ru_2_Cl_2_(μ-Cl)_2_(η^6^-*p*-cymene)_2_] with two thiosemicarbazones obtained by the condensation of N-(4-methoxybenzyl) thiosemicarbazide and 1,4-hydroxy-3-methoxyphenyl)ethan-1-one (**HL^1^**) or 2-fluoro-4-hydroxybenzaldehyde (**HL^2^**) was studied. The cationic complexes of formula [RuCl(η^6^-*p*-cymene)(HL)]^+^ were isolated as solid chloride and trifluoromethylsulfate (TfO) salts. A study of the solid state and NMR spectra suggests the presence in the material of two isomers that differ in the configuration in the iminic bond, C2=N3, of the coordinated thiosemicarbazone in the triflate salts and only the *E* isomer in the chloride. An X-ray study of single crystals of the complexes supports this hypothesis. The thiosemicarbazone ligand coordinates with the ruthenium center through the iminic and sulfur atoms to form a five-membered chelate ring. Furthermore, the isolation of single crystals containing the thiosemicarbazonate complex [Ru_2_(μ-L^2^)_2_(η^6^-*p*-cymene)_2_]^2+^ suggests the easy labilization of the coordinated chloride in the complex. The redox behavior of the ligands and complexes was evaluated by cyclic voltammetry. It seems to be more difficult to oxidize the complex derived from HL^1^ than HL^2^. The ability of the complexes to inhibit cell growth against the NCI-H460, A549 and MDA-MB-231 lines was evaluated. The complexes did not show greater potency than cisplatin, although they did have greater efficacy, especially for the complex derived from **HL^1^**.

## 1. Introduction

Thiosemicarbazones (TSCs) have shown a wide range of biological activities, including cytotoxic [1], antibacterial [2] and antiviral [3] properties. These compounds have a high affinity for metals due to the inclusion of a variety of different substituents and a wide range of possible coordination modes, which have been discussed in several reviews [4,5,6]. In addition, the biological properties of the ligands can be modified by variation of the substituents, and properties are frequently enhanced when they are coordinated with metal ions [7,8,9].

Ruthenium compounds are postulated to be more suitable substitutes for the antitumoral platinum family headed by cisplatin. In general, the former tend to produce fewer side effects, although the potency values and inhibition efficiency are usually lower. These claims are evidenced by several properties that confer cytotoxicity on these compounds that is comparable to that of platinum in conjunction with the avoidance of a significant proportion of the side effects. In general, the exchange kinetics of ruthenium complexes are similar to those of platinum, while the binding properties are similar to those of iron in a physiological environment by binding to proteins in the plasma such as serum albumina and transferrin [10]. Ruthenium has a rich redox chemistry that allows the formation of compounds in different oxidation states (mainly +II, +III and +IV), as well as the adaptation of the compound to the physiological environment [10]. Consequently, it is believed that ruthenium complexes, notably their arene derivatives, can participate in a variety of actions in the nucleus, mitochondria, and endoplasmic reticulum of cancer cells, stimulating apoptosis or autophagy processes and ultimately inhibiting angiogenesis, thus implying a very different therapeutic strategy when compared to cisplatin [11].

In the literature, a large number of arene complexes of Ru(II) have been described with different thiosemicarbazone ligands with the aim of increasing the cytotoxic activity in the resulting complex [12,13,14]. Studies on the antiparasitic [15,16,17,18] and antiviral [19] capacities have been carried out in the search for a synergistic action of the ruthenium–arene fragment on the properties of TSCs.

**Scheme 1:**
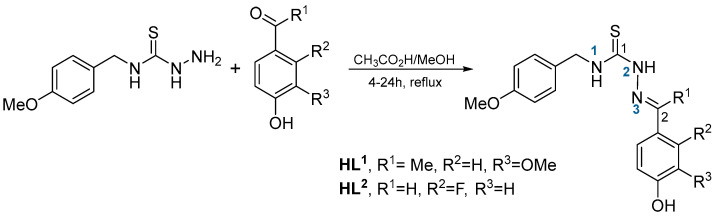
Synthesis of the thiosemicarbazone ligands.

In the work described here, we synthesized arene complexes of ruthenium(II) with two thiosemicarbazone ligands that contain substituents that impart biological activity to the molecules (Scheme 1) after metalation [20]. These compounds were structurally characterized and their redox activity and growth inhibition against several human tumor cell lines were determined. The results indicate that these newly synthesized Ru(II) complexes have promising biological activity.

## 2. Results and Discussion

The TSC ligands (**HL^1^** and **HL^2^**) were prepared in good yields by the condensation reaction of N-(4-methoxybenzyl) thiosemicarbazide and 1-(4-hydroxy-3-methoxyphenyl)ethan-1-one or 2-fluoro-4-hydroxybenzaldehyde in an equimolar ratio (Scheme 1) [21].

**Scheme 2:**
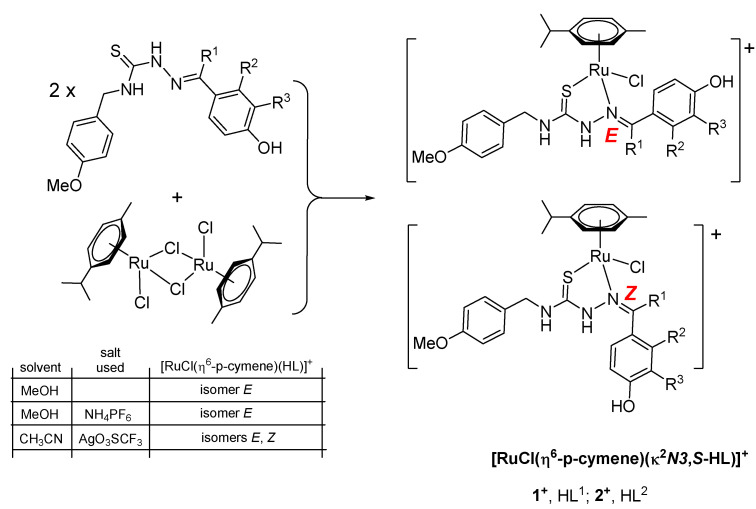
Synthesis of the thiosemicarbazone complexes. The two orientations possible for the substituent at C2 with respect to the C=N bond are depicted (resulting in the *E* and *Z* isomers).

The methods described previously for the preparation of thiosemicarbazone complexes with the fragment {Ru(η^6^-*p*-cymene)}^2+^ are highly varied [12,13,14,22,23,24,25]. As a consequence, a range of different media and conditions for the reaction between the ligands and the ruthenium precursor were explored. Specifically, the effects of the solvent used, the reaction rate, and the presence of certain anions in the medium were evaluated. The direct reaction of [Ru_2_Cl_2_(μ-Cl)_2_(η^6^-*p*-cymene)_2_] in methanol yielded the cationic complex [RuCl(η^6^-*p*-cymene)(κ^2^*N3*,*S*-HL^n^)]^+^. The complexes were isolated as chloride salts, and had different solubilities depending on the ligand. Complex **1(Cl)** was isolated as a precipitate from the reaction medium. However, ligand **HL^2^** seems to impart greater solubility on the complex, and in this case the reaction medium had to be concentrated to isolate the corresponding chloride (**2(Cl)**). An interesting aspect is that the ^1^H NMR spectra in dichloromethane suggest that the two materials contain the TSC ligand with a unique configuration at the C2=N3 bond, which we propose to be *E*, as in the free ligand (vide infra). In addition, when the reaction was carried out in the presence of NH_4_PF_6_, in contrast to the result previously reported by Su et al. [12] it did not lead to substitution of the anion or changes in the coordination mode of the thiosemicarbazone ligand. This finding suggests that the chloride anion must be strongly anchored to the complex cation by hydrogen bonds to the N1–H and N2–H groups (see Section 2.1). These bonds must strongly stabilize the [RuCl(η^6^-*p*-cymene)(*κ^2^N3,S*-HL)]Cl compounds, meaning that changes occur only by sequestering the chloride anion in the outer sphere of the complex. Thus, the corresponding cationic thiosemicarbazone complex (Scheme 2) could be isolated in the presence of several silver salts. However, the best yield was obtained when Ag(TfO) (silver triflate) was added to an acetonitrile solution of the ruthenium precursor; after ligand addition, the mixture was stored at room temperature under an inert atmosphere (Ar). The triflate salts of the complexes were then isolated in moderate yield as [RuCl(η^6^-*p*-cymene)(*κ^2^N3,S*-HL^n^)](TfO). These compounds are air-stable solids that are soluble in dichloromethane and methanol. The presence of signals attributable to the free ligand and precursor of ruthenium in the solutions in DMSO suggest the partial labilization of the complexes in this solvent. The NMR spectra are discussed in detail below; however, it is worth mentioning here that they unequivocally show the presence of ligands in both configurations.

The identities of the complexes **1(TfO)** and **2(TfO)** were verified by ^1^H NMR and IR spectroscopy and ESI mass spectrometry. The spectroscopic data and X-ray structure of single crystals indicate that ruthenium is coordinated to the TSC ligands through the iminic nitrogen (N3) and the sulfur atoms of the thiosemicarbazone as well as to a chloride ion and the η^6^-*p*-cymene ring (Scheme 2).

**Scheme 3:**
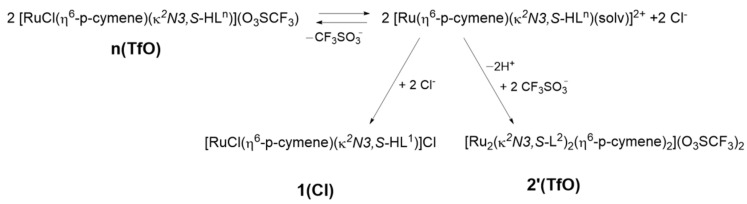
Possible processes for the formation of the complexes **1(Cl)** and **2′(TfO)** detected by X-ray diffraction.

Compound **1(TfO)** was crystallized under several sets of conditions and single crystals of **1(TfO)** and **1(Cl)** were obtained. The formation of the latter salt supports the idea of the high stability of the pair established between the cationic complex [RuCl(η^6^-*p*-cymene)(HL)]^+^ and the Cl^–^ anion but requires the labilization of the coordinated chlorine of the cationic complex **1^+^**. In contrast, crystallization of **2(TfO)** from methanol yielded single crystals of the dinuclear ruthenium thiosemicarbazonate complex [Ru_2_(η^6^-*p*-cymene)_2_(μ-*κ^2^N3,S*-L^2^)_2_](TfO)_2_ [**2′(TfO)**], in which the chloride ligand had been released and the TSC ligand deprotonated. Note that the formation of ruthenium(II) thiosemicarbazonate complexes was previously described and a relevant role was sometimes attributed to the (PF_6_^–^) anion [14,16]. However, Haribabu et al. attributed the formation of the thiosemicarbazonate complexes resulting from single deprotonation without the addition of base as a neutral monomer, [RuCl(L)(η^6^-*p*-cymene)]^0^, or dimer, [Ru_2_(η^6^-*p*-cymene)_2_(μ-*κ^2^N3,S*-L^2^)_2_]^2+^ [23], to the nature of the substituent on the thioamidic nitrogen N1 [19]. The results obtained for the TfO^–^ derivatives suggest the existence of several processes that affect the stability in solution. Our proposal is represented in Scheme 3, and is based on the following: (i) because this process was only observed in methanol, it is hypothesized that the possible role of triflate is to stabilize the intermediate complex; (ii) the formation of complex **1(Cl)** from solutions of previously isolated **1(TfO)** dissolved in methanol; (iii) the formation of **2′(TfO)** proves that the chloride ligand is spontaneously released from the cation **2^+^** to the reaction medium.

The analytical data are consistent with the proposed structures, and electrospray ionization (ESI) mass spectra showed *m*/*z* values and isotopic pattern distributions in agreement with those expected for the **HL^1^** and **HL^2^** ligands and their complexes (see Supplementary Materials). The (ESI)-MS for the **HL^1^**–**HL^2^** ligands showed signals due to |M + H|^+^. The spectra of **1(TfO)** and **2(TfO)** contain peaks due to |Ru(η^6^-*p*-cymene)(L^n^)|^+^ species, suggesting the mononuclear nature of these compounds and the lability of the Ru–Cl bond.

The IR spectra of the free ligands contain a band at 1536–1460 cm^−1^ corresponding to the ν(C=N) vibration. After coordination, a shift to higher frequencies in the range 1561–1459 cm^−1^ is observed for both **1(TfO)** and **2(TfO)** complexes. Moreover, the ν(C=S) band shifts to lower wavenumbers (818–816 cm^−1^) with respect to the free ligand (845 cm^−1^), which is consistent with the bidentate *S,N*-coordination of the thiosemicarbazone [20,26,27].

In the ^1^H NMR (CD_2_Cl_2_) spectra of the ligands, the azomethinic proton (=N–NH) signals appear at 8.66 (**HL^1^**) and 8.97 ppm (**HL^2^**). The methyl substituent at C2 (N3=C2CH_3_) provides a signal at 2.25 ppm (**HL^1^**), while the C2–H proton (N3=C2H) is observed at 7.91 ppm (**HL^2^**). The same protons in the complexes are deshielded due to a coordination effect (Table 1), which confirms the *S*,*N3*-coordination mode of the thiosemicarbazone [14,28,29,30].

However, a significant feature of the ^1^H NMR spectra of the ruthenium complexes is that there are two different sets of signals (including those of the *p*-cymene group) corresponding to the *E* and *Z* isomers, which are related to the arrangement of the C2=N3 bond [14]. For the sake of simplicity, the same notation, *Z* and *E*, is used to identify both the type of isomer/configuration (for example, on the C2–N3 link) and the rotamer/conformer (as derived from the substituents on the N2–C1 link) along the thiosemicarbazone/-ate arm C2–N3–N2–C1(S)–N1).

The spectra of the triflate derivatives **1(TfO)** and **2(TfO)** is discussed first. The ^1^H-NMR spectrum of **1(TfO)** (with a ratio of around 60:40 between the sets of signals) contains a doublet for each of the four *p*-cymene ring protons, two doublets for the methyl groups of isopropyl moieties (indicating their lack of equivalence), and one singlet for the methyl groups [26]. The four proton resonances attributed to the *p*-cymene ring in CD_2_Cl_2_ are in the ranges 5.35–4.01 ppm (*E* isomer) and 5.63–4.60 ppm (*Z* isomer). One of the four proton resonances attributable to *p*-cymene is shifted to higher field at 4.01 ppm in the *E* isomer. This is probably due to the proximity of the 4-hydroxy-3-methoxyphenyl group of the TSC in this configuration. However, in the *Z* isomer the resonances assigned to the *p*-cymene ring are shifted downfield compared to the *E* isomer. Two singlets at 1.99 and 2.26 ppm correspond to the methylene protons of the *E* and *Z* isomers, respectively. The complexity of the signals of the *p*-cymene group is due to the asymmetry of the complex. With respect to the TSC ligand, the methyl protons of N3=C2–CH_3_ are observed at 2.74 ppm (*E*) and 2.98 ppm (*Z*), with the high-field shift of the latter signal possibly due to an interaction between methyl groups of the ligand (N3=C2–*CH_3_*) and the methyl group of *p*-cymene in the *Z* isomer [23]. The multidimensional spectra support the proposed structures for all complexes. In particular, the NOESY spectra confirm the presence of the *E*/*Z* arrangement. The presence of the *E* isomer in the single crystal of **1(TfO)** and the *Z* isomer in **1(Cl)** confirms the role of this interaction (vide infra).

The ^1^H NMR (CD_2_Cl_2_) spectrum of **2(TfO)** contains two different sets of signals corresponding to the *E*/*Z* isomers (in a similar ratio to **1(TfO)**) of the bidentate ligand coordinated to the metal center. The iminic protons show peaks at 10.62 (*E*) and 12.10 (*Z*) ppm. The low-field shift in the signal for the *Z* isomer is attributable to the interaction between the iminic proton of the ligand and one of the aromatic protons of the *p*-cymene. The {^1^H-^1^H}NOESY experiment confirms this interaction in the *Z* isomer. 

Three signals of different intensity are observed in the ^19^F{^1^H} NMR spectrum of **2(TfO)**: one at −70.13 ppm, corresponding to the [TfO]^−^ anion, and two others corresponding to HL^2^ at −107.27 and −110.68 ppm. These signals are consistent with the presence of the two different species (*E* and *Z*).

The spectroscopic characterization experiments on complexes **1(Cl)** and **2(Cl)** were mainly carried out on materials obtained by synthesis in the presence of NH_4_PF_6_ (see Section 4). In all cases the ^31^P NMR spectra did not contain any signal, and therefore the presence of the PF_6_^–^ anion can be ruled out. On comparing the ^1^H NMR data for these complexes, it can be observed that the signals corresponding to the *p*-cymene groups and substituents of the TSC chain are practically identical to those observed for the *E* isomer of the triflate derivatives (Table 1). One exception concerns the signals due to the N–H groups, which are markedly more deshielded (in the case of N2–H, by around 2 ppm) with respect to the isomer of the triflate group. This finding suggests that the H-bonding observed in the X-ray structure of **1(Cl)** (vide infra) is present in **2(Cl),** and is maintained in dichloromethane solutions as well.

### 2.1. Crystal Structures

**Figure 1:**
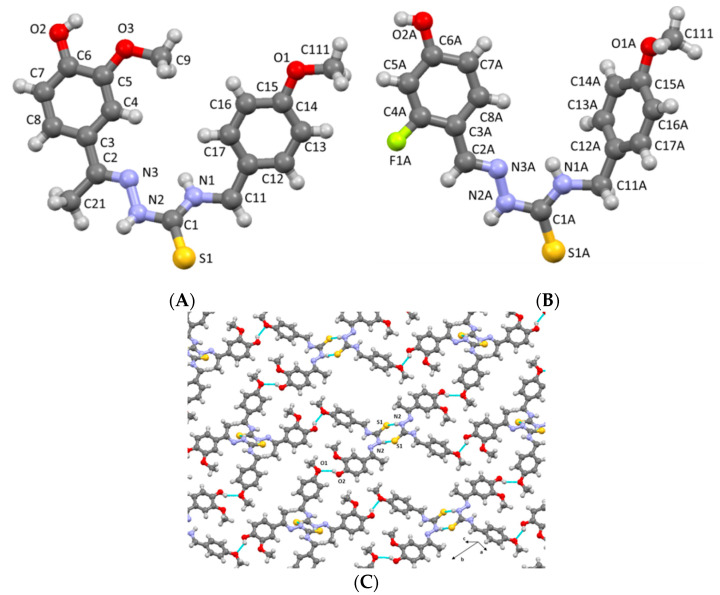
The molecular structures of **HL^1^** (**A**) and **HL^2^** (**B**), where only one of the two molecules present in the asymmetric unit is included, and the intermolecular association in **HL^1^** (**C**).

The molecular structures of the free ligands **HL^1^** and **HL^2^** are depicted in Figure 1, and a selection of the most relevant structural data is included in Table 2. Although both structures are very similar, with the same conformation along the thiosemicarbazone arm (*E*,*E*,*E*,*Z*), a few differences can be highlighted. The S1–C1 distance is slightly longer in **HL^2^** than **HL^1^**, although both values are within the range expected for an S=C double bond. As usually observed in metal-free thiosemicarbazones, the N2–C1 distance is slightly shorter than that expected for a single N–C bond (for example, the N1–C11 bond), and is statistically equivalent in both structures. Nevertheless, N2–N3 is slightly shorter in **HL^2^** than in **HL^1^**. In general, the values observed in the thiosemicarbazone chain are consistent with the existence of π delocalization along the whole chain.

The **HL^1^** and **HL^2^** molecules associate in a similar way in the crystal. In both cases, N2–H…S1 interactions are established to form centrosymmetric pairs (synthon *R*_2_^2^(8)). These dimers in turn form 2D associations through hydrogen bonds involving the OH groups of the substituent on carbon C2 and the MeO group (in **HL^1^** in Figure 1C) or S1 (**HL^2^**) as acceptors.

**Figure 2:**
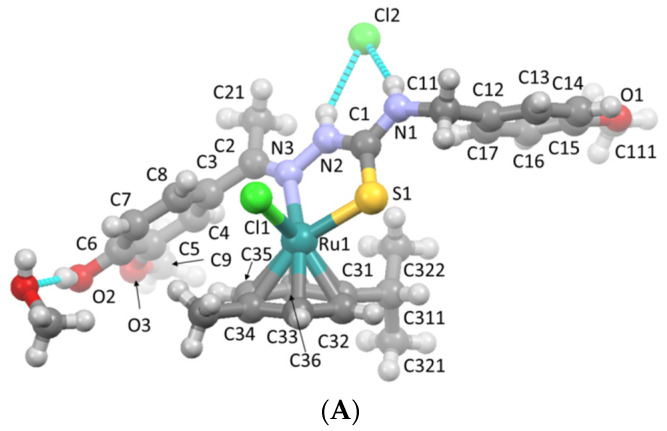

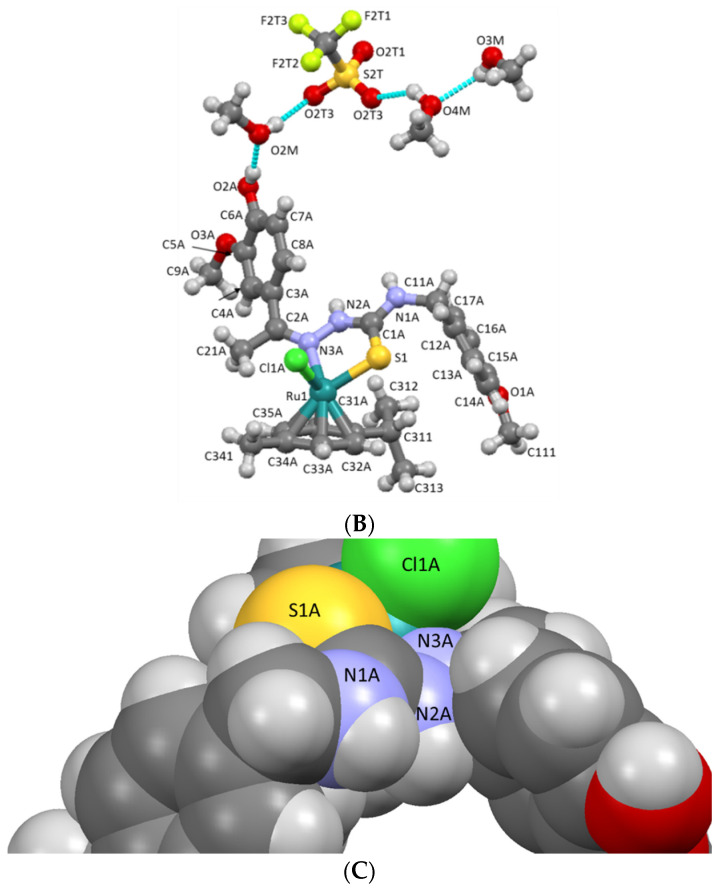
(**A**) The asymmetric unit in the structure of **1(Cl)**.CH_3_OH. (**B**) representation of one of the molecules of complex observed in **1(TfO)**.2(CH_3_OH), showing the H-bonding between ions and solvent molecules. (**C**) Space-filling model of the molecule of **1(TfO),** showing that the *Z* configuration of the C2=N3 bond hinders the H-bonding role of the groups N1–H, in particular N2–H.

The structures of **1(Cl)**.CH_3_OH and **1(TfO)**.2(CH_3_OH) are shown in Figure 2 as representative examples. In these structures, the cationic complex [RuCl(η^6^-*p*-cymene)(HL^1^)]^+^ (**1^+^**) appears and forms crystals with chloride and trifluoromethanesulfonate anions. Single crystals of **1(Cl)** were obtained as hydrate and methanol solvate phases (see Section 4). These crystals have statistically equivalent structural parameters, and we therefore only include the data corresponding to the methanol-solvated system. The crystal structure of the triflate derivative contains two **1(TfO)** units per asymmetric unit (identified as molecules A and B in Table 2). Although relevant differences were not observed regarding the cationic unit **1^+^**, as described below, and it is similar to those found in other [RuCl(η^6^-*p*-cymene)(HL)]^+^ complexes [14,15,16,23], its interaction with the solvent molecules (MeOH) does imply crystallographic differences. Unfortunately, the structural data for this compound have standard deviation values that limit the conclusions that can be drawn, specifically when comparing the two types of structures, i.e., that of the complex cation with the free ligand **HL^1^**.

In the structures of both cations **1^+^**, the ligand **HL^1^** is coordinated to ruthenium by the sulfur atom and the nitrogen N3 to form a five-membered chelate ring. The pseudo-octahedral (piano stool-shaped) coordination around the ruthenium atom is completed by the *para*-cymene ring and the chloride ligand.

In order to achieve κ^2^-*S*,*N*3 coordination, the conformation of the N2–C1 bond in the ligand must change from *E*, as observed in the free ligand, to *Z*; in the determination of the conformers of the complexes, the metal–ligand binding is ignored. However, an interesting difference between the two cations is the conformation of the C2=N3 bond of the **HL^1^** ligand. In the chloride derivative the configuration of this bond is *E*, which directs the vanillin ring towards the ruthenium atom (Figure 2A); however, in the crystal structure of the triflate derivative the conformation is *Z*, and the methyl group is oriented towards the metal (Figure 2B). This arrangement explains the differences in the Ru–S1 and Ru–N3 distances observed in the two compounds: the former is clearly shorter, while the latter is clearly longer in the chloride than in the triflate. Bearing in mind the greater steric hindrance that the vanillin ring must impose, it stands to reason that the approach of the **HL^1^** ligand must be facilitated by the more distant sulfur. This proposal is supported by the C1–S1–Ru1 angle in the *E*-isomer, as observed in **1(Cl)**, which is wider than in the *Z*-isomer observed in **1(TfO)**. Similarly, the S1–Ru1–N3 chelate angle is wider in the chloride derivative (82.30(4)°) than in the triflate (80.99(12) and 81.09(12)°).

Finally, another interesting difference between the two isomers of the cationic complex **1^+^** is that the Ru1–Cl1 distance is clearly longer in the *E*-isomer (2.4238(4) Å) than in the *Z*-isomer (2.3997(14) and 2.4015(14) Å).

There do not seem to be significant differences in the binding of ruthenium to the *para*-cymene ring: the Ru1–C distances range from 2.1747(16) to 2.2644(16) Å (average value 2.208(1) Å) in the *E*-isomer and 2.178(5)–2.261(6) Å for Ru1–C and 2.173(6)–2.268(6) Å for Ru2–C (average values 2.208(2) and 2.206(2) Å, respectively) in the *Z*-isomer. In both cases the shortest Ru–C distance is observed for the interaction with the carbon ortho to the isopropyl (C36) substituent, and the distance to the centroid of the *para*-cymene ring is statistically equivalent at 2.208(1) Å for the *E*-isomer and 2.208(2) and 2.206(2) Å for the *Z*-isomer. However, as can be seen in Figure 2B, in the *Z* conformation the methyl groups of the thiosemicarbazone and *para*-cymene are oriented convergently. This observation reinforces the interaction evidenced in the NMR spectrum between the two groups as well as the assignment of each isomer in the spectra (vide supra).

Coordination of the neutral thiosemicarbazone ligand does not usually produce large changes in bond distances in the thiosemicarbazone chain. Furthermore, when differences between the bond distances in complexes are observed, the degree of uncertainty associated with these values often makes it difficult to draw clear conclusions. This is the case for the N2–N3 distances, which should not be considered as statistically different when comparing the spectrum of **HL^1^** and the values determined for the two **1^+^** isomers. Similarly, the standard deviation values do not allow us to conclude that there is a lengthening of the S1–C1 distance in coordinated **HL^1^** in any example, although there is a shortening of the N2–C1 distance and a lengthening of N3–C2 in the *E*-isomer (Table 2).

As mentioned above, the material isolated in the synthesis of complex **1(TfO)** contains a mixture of the *E*/*Z* isomers. It is therefore interesting to consider the factors that could induce the crystallization of one or other isomer; in this respect, the type of intermolecular association is probably one of the most relevant factors.

**Figure 3:**
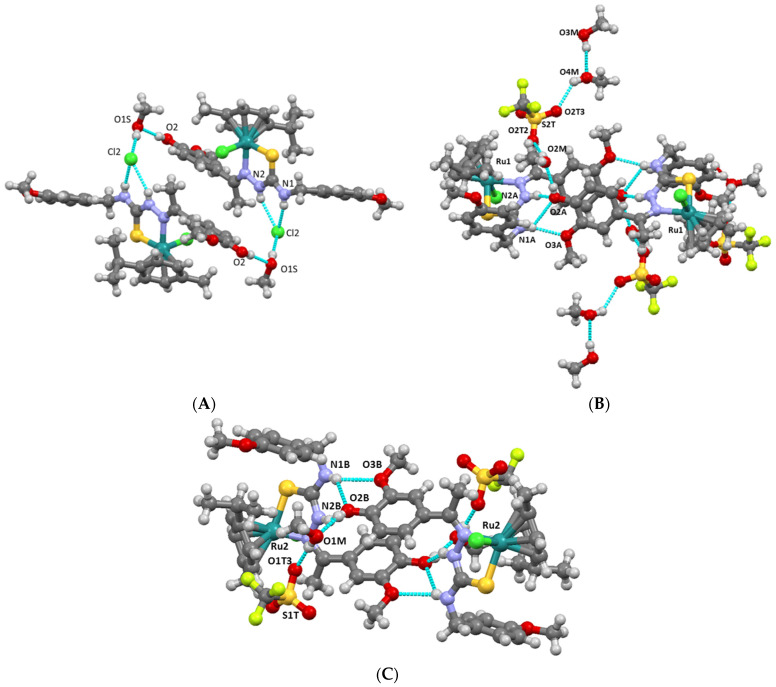
The intermolecular associations in **1(Cl)**.CH_3_OH (**A**) and **1(TfO)**.2(CH_3_OH) (**B**,**C**).

The association in the **1(Cl)**.CH_3_OH crystal is dominated by the cationic nature of the complex and the presence of a methanol molecule. Two hydrogen bonding interactions (N1–H…Cl2 and N2–H…Cl2) form a six-membered ring (usually described as chelated hydrogen bonds or synthon *R*_2_^1^(6)), and the **1^+^** cation is associated with the Cl^−^ anion. The interatomic distances and angles are almost equivalent (Table S2), indicating the presence of a moderate H-bond [31]. Two of these ionic pair units are in turn associated through a hydrogen bond involving a methanol molecule that interacts with the Cl2 and the O2–H donor (Figure 3A).

Note that the second interaction (N2–H…Cl2) and the association of the ion pairs **1(Cl)** mediated by methanol can hardly be established in the case where the ligand acquires the *Z* conformation at the C2=N3 bond, as can clearly be seen in Figure 2C.

In the crystal structure of **1(TfO)**.2(CH_3_OH), the cationic complex is associated with an anion, in this case with an intervening methanol molecule. These units associate with another crystallographically identical unit through a combination of three-center hydrogen bonding and chelation involving the N2–H and N1–H groups of one molecule and the O3 and O2 of its partner (Table S2). Interestingly, the crystallographically different molecules do not interact with each other or through solvent molecules (Figure 3B,C).

In addition, the triflate anion has a level of disorder; this was interpreted by introducing two alternative positions for this anion in the model. It was observed that the role of the N2–H and N1–H groups in the association with the oxygen atoms of the neighboring vanillin group in this case is much weaker than in **1(Cl)**, as indicated by the structural parameters (Table S2).

**Figure 4:**
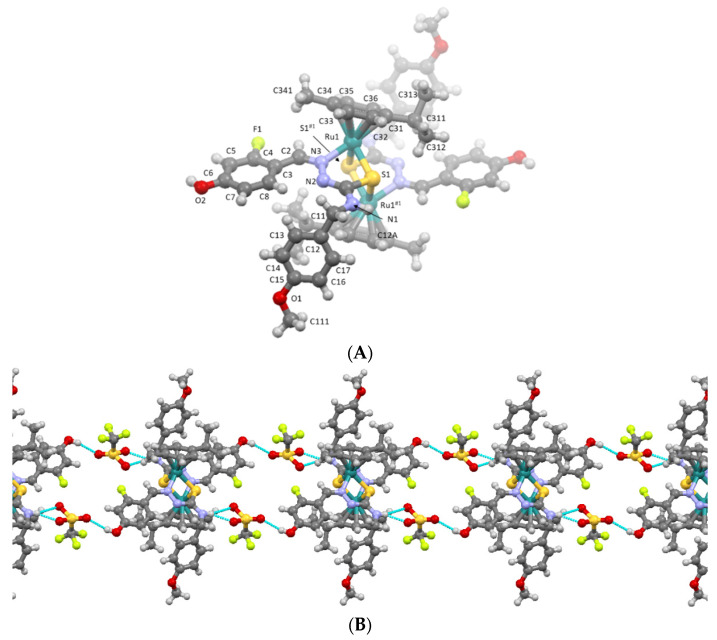
The X-ray structure of the cation in [Ru_2_(*p*-Cm)_2_(μ-L^2^)_2_][O_3_SCF_3_]_2_ (**A**) and the molecular association based on H-bonding in the crystal (**B**).

In the structure of [Ru_2_(μ-L^2^)_2_(η^6^-*p*-cymene)_2_][O_3_SCF_3_]_2_, **2′(TfO)**, the thiosemicarbazone ligand undergoes deprotonation and the sulfur atom acts as a bridge, displacing the chloride ligand from coordination to ruthenium (Figure 4A). The resulting thiosemicarbazonate coordination mode μ-κ^2^S,N3:κS, which has been previously observed in other d^6^ systems such as ruthenium(II) [17,19] and rhenium(I) [32], gives rise to dimeric structures based on the formation of Ru_2_S_2_ diamonds. Note that, as observed in the rhenium complexes, dimers based on M_2_S_2_ can be formed with the thiosemicarbazonate ligand through the formation of a four-membered chelate ring by coordination of nitrogen N2 and the sulfur [12]. Although several arrangements are possible, in the present case the mass center of the dimer lies on an inversion center, which means that the dimer consists of two identical units. The Ru1–S1^#1^ bridging bond (2.4085(9) Å) is substantially longer than the bond that forms the chelate ring (2.3617(10) Å). The conformation of the thiosemicarbazonate chain (C2–N3–N2–C1(S)–N1) is *Z*,*E*,*Z*,*E*, where the substituents on the C2 and N1 atoms seem to avoid the coordination core.

The Ru–S1 distance is intermediate between those observed in **1(Cl)** and **1(TfO),** while the Ru1–N3 distance is clearly the shortest of all the ruthenium complexes included in this study. When compared to the free and non-deprotonated **HL^2^** ligand structure, the S1–C1 distance seems to be much longer, while N2–C1 is much shorter. The same conclusion can be drawn for the **HL^1^** derivatives (Table 2). On the other hand, the S1–Ru1–N3 chelate angle is clearly more closed compared to those in the thiosemicarbazone derivatives of **HL^1^**.

The Ru1–C distances, as in the derivatives of **HL^1^**, range between 2.184(5) and 2.267(5) Å; again, the shortest distance is that established with one of the carbons in the ortho position to the isopropyl substituent, although the distance from ruthenium to the centroid of the aromatic ring is much shorter (1.4728(3) Å) than in the previously discussed compounds.

The complex cation **(2′)^2+^** associates with the triflate anion through hydrogen bonds involving the donor groups N1–H and O2–H and the oxygen atoms of the triflate anions. These interactions lead to the anions and cations being arranged in chains, as shown in Figure 4B.

### 2.2. Cyclic Voltammetry Results

The redox behavior of the ligands and Ru(II) complexes was studied in methanol solution by cyclic and square wave voltammetry at a platinum working electrode.

**Figure 5:**
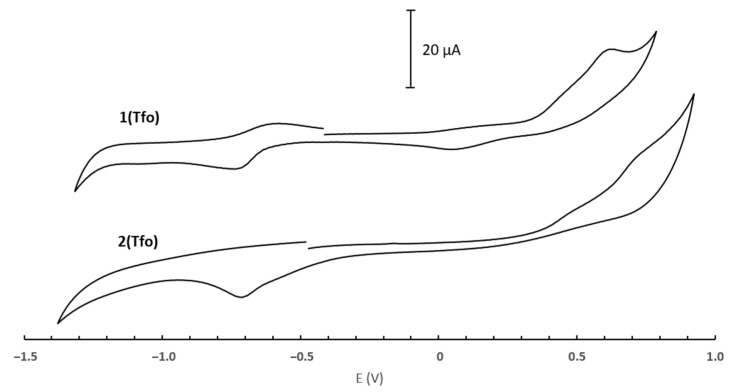
Cyclic voltammograms of complexes measured in CH_3_OH (1 mM solutions) with 0.1 M TBAP as the supporting electrolyte. Scan rate: 200 mV/s.

The electrochemical data are summarized in Table 3, and representative voltammograms of complexes are shown in Figure 5.

The free ligands **HL^1^** and **HL^2^** showed one irreversible oxidation wave at 0.59 and 0.58 V and one reduction wave at −0.75 and −0.8 V, respectively. The oxidation waves can be attributed to the redox behavior of the imine group present in the ligands [18,33].

Both processes are irreversible in the scan rate range 0.05–1 V/s, indicating that the electrochemically generated products are not stable. Reduction waves were not observed in the first cycle of the negatively initiated voltammograms or when the scan was reversed before ligand oxidation. These findings confirm that only the products previously generated in the anodic scan are available for reduction.

The effect of the scan rate on the electrochemical response of the ligands was investigated between 0.05 and 1 V/s. The current was found to be directly proportional to the square root of the scan rate, indicating the diffusion-controlled nature of the processes.

The voltammograms of the complexes have different features from those described for the corresponding ligands, which suggests that the metal center has an effect on the redox properties of the compounds.

In the scan towards the anodic side of the voltammograms of complexes **1(TfO)** and **2(TfO),** signals can be observed close to the solvent and electrolyte discharge limit that make their observation less clear. Both complexes undergo two overlapped irreversible oxidations and one reduction within the potential window of the CH_3_OH/TBAP supporting electrolyte (Figure 5). The first oxidation response is a poorly defined wave at 0.460 for **1(TfO)** and 0.490 V for **2(TfO)**. However, this wave has better definition in the square wave voltammetry experiments (see Supplementary Materials). The second oxidation response is found at 0.630 and 0.720 V for **1(TfO)** and **2(TfO)**, respectively.

After scan reversal towards negative potentials, both complexes experience a similar voltammetric response at scan rates >0.2 V/s. A quasi-reversible reduction peak was observed with cathodic peak potentials at −0.71 and −0.73 V for **1(TFO)** and **2(TFO)**, respectively. The anionic species formed after reductions are relatively stable at high scan rates (see Supplementary Materials). However, despite the fact that the ratio between cathodic and anionic current intensities clearly increases with the scan rate, it never reaches unity under the experimental conditions employed. Additionally, peak-to-peak separations for the ruthenium complexes are larger than those of the internal ferrocene standard, a finding that indicates slow electron transfer kinetics for the ruthenium complexes.

For both ligands and complexes, it was observed that the current intensities of the signals increased with scan rate. A linear dependence of peak current on the square root of the scan rate is clearly observed, showing the diffusion-controlled nature of the oxidoreduction processes involving the complexes.

The oxidation and reduction potentials of complexes **1(TFO)** and **2(TFO)** seem to be particularly sensitive to the R^1^ (H/CH_3_) substituent on the imine carbon of the thiosemicarbazone. For example, complex **2(TFO)**, in which R^1^ = H, has higher oxidation and reduction potentials than complex **1(TFO)** (R^1^ = CH_3_), although it is probable that the other substituents R^2^ and R^3^ influence the potential as well.

### 2.3. Cytotoxicity Assays

Preliminary studies of the cytotoxicities of **1(TfO)** and **2(TfO)** and their corresponding free ligands were evaluated by means of assays in the human cancer cell lines NCl-H460, A549, and MDA-MB-231 (see caption in Table 4). The ligand **HL^2^** showed low cell growth inhibition (<50%) at the maximum concentration tested (100 mM) for all three cell lines, while **HL^1^** showed cell growth inhibition >50% in A549 and MDA-MB-231 cells. Similarly, **1(TfO)** showed relevant cell growth inhibition for A549 and MDA-MB-231 cells; therefore, it was possible to generate cell growth inhibition curves in order to calculate IC_50_ values for both compounds. Although the cytotoxic activity in absolute terms is weaker for **HL^2^** and its complex, it can be observed that in this case the activity of the complex **2(TfO)** against the A549 cell line increases substantially, allowing its IC_50_ to be determined as well.

Taking into account the dissociation of the two complexes in DMSO, it is probable that the cell growth inhibitory activity has contributions from several species generated in the biological medium [34]. In general, however, a slight increase in the activity upon metalation of the two ligands was observed.

It is interesting to note that the IC_50_ values of both complexes and the ligand **HL^1^** are higher than those of cisplatin (Table 4), which means that the necessary dose must be higher to reduce the number of cells by half, while the efficacy of the inhibition (referred to the maximum inhibition percentage achieved) is greater for **1(TfO)** in the A549 and MDA-MB-231 cell lines.

We consider the results obtained to be promising in terms of the antitumor activity of the compounds because, although the inhibitory power is not superior to that of cisplatin, the inhibitory efficacy of **1(TfO)** is comparable to that of the reference.

## 3. Conclusions

The thiosemicarbazones and the arene-complexes of ruthenium(II) show an interesting capacity for cell growth inhibition, and there is increasing interest in the possible potentiation that may be inherent in complexes formed by two fragments. As a consequence, a growing number of papers have been published on the reaction of two-fragment species using varied methodologies, and numerous different types of final product have been described. The work reported here concerned the reaction of two thiosemicarbazones with potential biological activity with the {RuCl(η^6^-*p*-cymene)}^+^.

In general, the stoichiometry of the isolated complex does not seem to depend on the counterion used, and the isolated product is always constituted by a salt of the cation [RuCl(η^6^-*p*-cymene)(κ^2^N3,S-HL)]^+^. However, the geometry of the coordinated ligand is affected by the nature of the anion present. Specifically, the chloride ion, probably through the establishment of a double hydrogen bond (N2–H…Cl and N1–H…Cl) with the coordinated TSC, favors the presence of a single geometric isomer (*E*) with respect to the double bond C2=N3. When the chloride is removed from the second coordination sphere, the ligand can adopt the two configurations for the C2=N3 bond, and the NMR spectra show an almost equal population of the two isomers. The cationic complexes show a tendency to liberate the coordinated chloride, and in the absence of base and at room temperature, the dimeric complex [Ru_2_(μ-κ^2^N3,S-L)_2_(η^6^-*p*-cymene)_2_(HL)_2_]^2+^ can be isolated.

A study of the complexes suggests that the substituent in the iminium group affects the electrochemical properties of the complex, with the aldehyde derivatives (R^1^ = H) having higher oxidation and reduction potentials than the ketone derivatives (R^1^ = CH_3_).

Finally, while the complexes show lower cytotoxic activity than cisplatin as measured by inhibition potency (higher IC_50_), but the maximum percentage inhibition of the **HL^1^**-derived complex against A549 and MDA-MB-231 cell lines is comparable to that of the standard. Further work is currently underway in our laboratory aimed at modifying the types of substituents on the thiosemicarbazone chain with the aim of improving the stability of the complex and the inhibitory power against cell growth.

## 4. Experimental Section

### 4.1. General Procedures

Solvents were purified by distillation from the appropriate drying agents [35] and were degassed before use. All reagents were obtained from commercial sources and were used without further purification.

N-(4-Methoxybenzyl)thiosemicarbazide (MeO-TSC) [21] and the starting [Ru_2_Cl_2_(μ-Cl)_2_(η^6^-*p*-cymene)_2_] [36] were prepared following previously published methods. NMR spectra were recorded in CD2Cl2 or DMSO at room temperature on a Bruker ARX 400 instrument with resonating frequencies of 400 MHz (^1^H), 376 MHz (^19^F{^1^H}) and 100 MHz (^13^C{^1^H}) using the solvent as the internal lock. ^1^H and ^13^C{^1^H} signals were referred to internal TMS and CFCl_3_ for ^19^F{^1^H}; downfield shifts (expressed in ppm) were considered positive. ^1^H and ^13^C{^1^H} NMR signal assignments were confirmed by {^1^H,^1^H} COSY, {^1^H,^13^C} HSQC, {^1^H,^13^C} HMBC, and {^1^H,^1^H} NOESY experiments. Coupling constants are provided in Hertz. Infrared spectra were obtained on a Jasco FT/IR–6100 spectrophotometer. C and H analyses were carried out on a Carlo Erba 1108 analyzer.

Melting points were determined on a Gallenkamp MFB-595 apparatus, and were not corrected. Mass spectra were recorded on a microTOF-Focus (Bruker Daltonics- Bruker Scientific Instruments, Bremen, Germany) mass spectrometer operating under ESI conditions for ligands and complexes.

### 4.2. Synthesis of Thiosemicarbazone (TSC) Ligands (**HL^1^** and **HL^2^**)

#### 4.2.1. Preparation of N-(4-Methoxybenzyl)-(3-methoxy-4-hydroxybenzophenone) Thiosemicarbazone (**HL^1^**)

Thiosemicarbazide MeO-TSC (500 mg, 2.36 mmol) and 1,4-hydroxy-3-methoxyphenyl)ethan-1-one (395 mg, 2.36 mmol) were dissolved in MeOH (20 mL) with 1–2 drops of acetic acid. The resulting mixture was stirred and heated under reflux. After 24 h the mixture was allowed to cool to room temperature. The solvent was partially evaporated (5 mL) and the resulting white solid was filtered off and dried under vacuum over CaCl_2_/KOH. Crystals suitable for X-ray analysis were obtained by slow evaporation of a solution of MeOH at room temperature.

Yield: 672 mg, 79%. Mp. 167–170 °C Anal. calcd. for C_18_H_21_N_3_O_3_S (359.13): C: 60.15, N: 11.70, H: 5.89, S: 8.90%; Exp. C: 60.88, N: 12.02, H: 5.90, S: 8.40%. MS-ESI [*m*/*z* (%)]: 360.14 (100) [M + H]^+^. IR (ATR, ν/cm^−1^): 3167 br (NH, OH), 1596 s (C=N), 845 m (C=S).

**Scheme 4:**
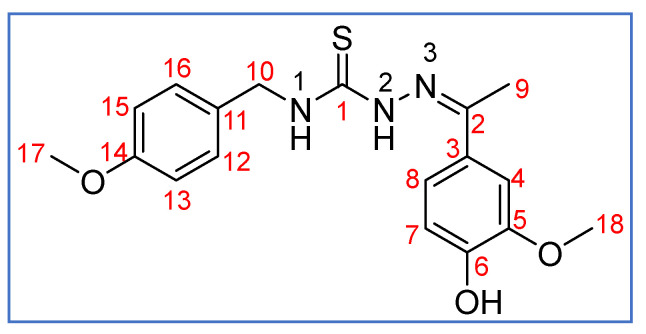
Atomic numbering scheme for **HL^1^**.

^1^H NMR (400 MHz, DMSO-*d*_6_, ppm, see Scheme 4 for the atomic numbering used): 10.22 (s, 1H, N^2^-H), 9.38 (s, 1H, OH), 8.80 (t, 1H, ^3^*J*_H-H_ = 6.2 Hz, N^1^-H), 7.43 (m, 1H, C^4^-H), 7.34 (dd, 1H, ^3^*J* _H-H_ = 8.3 Hz, ^4^*J* _H-H_ = 2.1 Hz, C^8^-H), 7.31 (m, 2H, C^12^-H, C^16^-H), 6.91 (m, 2H, C^13^-H, C^15^-H), 6.77 (d, 1H, ^3^*J* _H-H_ = 8.3 Hz, C^7^-H), 4.78 (d, 2H, ^3^*J*_H-H_ = 6.1 Hz, C^10^-H), 3.81 (s, 3H, C^18^-H), 3.73 (s, 3H, C^17^-H), 2.27 (s, 3H, C^9^-H). ^1^H NMR (400 MHz, CD_2_Cl_2_-*d*_2_, ppm): 8.66 (s, 1H, N^2^-H), 7.85 (s, br, 1H, N^1^-H), 7.31 (m, 2H, C^12^-H, C^16^-H), 7.22 (d, 1H, ^3^*J* _H-H_ = 2.1 Hz, C^8^-H), 7.19 (dd, 1H, ^3^*J* _H-H_ = 8.3, ^4^*J* _H-H_ = 2.1 Hz, C^7^-H), 6.89 (m, 2H, C^13^-H, C^15^-H), 5.85 (s, 1H, C^4^-H), 4.85 (d, 2H, ^3^*J*_H-H_ = 5.7 Hz, C^10^-H), 3.85 (s, 3H, C^18^-H), 3.78 (s, 3H, C^17^-H), 2.25 (s, 3H, C^9^-H). ^13^C{^1^H} NMR (100 MHz, DMSO-*d*_6_, ppm): 178.03 (s, C^1^), 158.24 (s, C^14^), 148.70 (s, C^2^), 148.31 (s, C^3^), 147.38 (s, C^5^), 131.24 (s, C^11^), 128.94 (s, C^6^), 128.66 (s, C^12^, C^16^), 120.26 (s, C^8^), 115.05 (s, C^7^), 113.61 (s, C^13^, C^15^), 110.71 (s, C^4^), 55.80 (s, C^18^), 55.06 (s, C^17^), 46.23 (s, C^10^), 14.16 (s, C^9^).

#### 4.2.2. Preparation of N-(4-Methoxybenzyl)-(2-fluoro-4-hydroxybenzaldehyde)-thiosemicarbazone (**HL^2^**)

This ligand was obtained by following a procedure similar to that for **HL^1^**, except using thiosemicarbazide MeO-TSC (200 mg, 0.95 mmol) and 2-fluoro-4-hydroxybenzaldehyde (133 mg, 0.95 mmol). Crystals suitable for X-ray analysis were obtained by slow evaporation of a solution in MeOH at room temperature.

Yield: 253, 80%. Mp. 213–214 °C. Anal. calcd. C_16_H_16_FN_3_O_2_S 1/3 H_2_O (339.38): C: 56.62, N: 12.38, H: 4.95, S: 9.44%; Exp. C: 56.73, N: 11.86, H: 5.34, S: 9.14%. MS-ESI [*m*/*z* (%)]: 334.10 (100) [M + H]^+^. IR (ATR, ν/cm^−1^): 3181 br (NH, OH), 1623 s (C=N), 846 m (C=S).

**Scheme 5:**
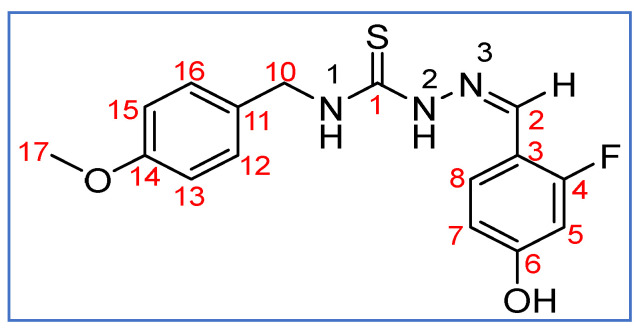
Atomic numbering scheme for **HL^2^**.

^1^H NMR (400 MHz, DMSO-*d*_6_, ppm, see Scheme 5 for the atomic numbering used): 11.52 (s, 1H, N^2^-H), 10.41 (s, 1H, OH), 8.95 (t, 1H, ^3^*J* _H-H_ = 6.2 Hz, N^1^-H), 8.20 (s, 1H, C^2^-H), 8.04 (t, 1H, ^3^*J* _H-H_ = ^4^*J* _H-F_ = 8.8 Hz, C^8^-H), 7.29 (d, 2H, ^3^*J* _H-H_ = 8.7 Hz, C^12^-H, C^16^-H), 6.89 (d, 2H, ^3^*J* _H-H_ = 8.7 Hz, C^13^-H, C^15^-H), 6.65 (dd, 1H, ^3^*J* _H-H_ = 8.7, ^4^*J* _H-H_ = 2.3 Hz, C^7^-H), 6.59 (dd, 1H, ^3^*J* _H-F_ = 12.7, ^4^*J*_H-H_ 2.3 Hz, C^5^-H), 4.75 (d, 1H, ^3^*J* _H-H_ = 6.2 Hz, C^10^-H), 3.73 (s, 3H, C^17^-H). ^1^H NMR (400 MHz, CD_2_Cl_2_-*d*_2_, ppm): 8.97 (s, 1H, N^2^-H), 7.91 (s, 1H, C^2^-H), 7.68 (t, ^3^*J* _H-H_ = *J*^4^_H-F_ = 8.5 Hz, 2H, C^8^-H, N^1^-H), 7.32 (d, 2H, ^3^*J* _H-H_ = 8.7 Hz, C^12^-H, C^16^-H), 6.89 (m, 2H, C^13^-H, C^15^-H), 6.65 (dd, 1H, ^3^*J* _H-H_ = 8.6 Hz, ^4^*J*_H-H_ = 2.5 Hz, C^7^-H), 6.60 (dd, 1H, ^3^*J* _H-F_ = 11.8 Hz, ^4^*J*_H-H_ = 2.4 Hz, C^5^-H) 4.85 (d, 2H, ^3^*J* _H-H_ = 5.9 Hz, C^10^-H), 3.79 (s, 3H, C^17^-H). ^13^C{^1^H} NMR (100 MHz, DMSO-*d*_6_, ppm): 177.06 (s, C^1^), 161.8 (s, d, ^1^*J* _C-F_ = 249Hz, C^4^), 160.07 (d, ^2^*J*_C-F_ = 12 Hz, C^3^), 158.20 (s, C^14^), 135.30 (d, ^3^*J*_C-F_ = 4.3 Hz, C^2^), 131.40 (s, C^11^), 128.65 (s, C^12^, C^16^), 127.73 (d, ^3^*J*_C-F_ = 4.6 Hz, C^8^), 113.57 (s, C^13^, C^15^), 112 (d, ^4^*J*_C-F_ = 2.5 Hz, C^7^), 102.33 (d, ^2^*J*_C-F_ = 23 Hz, C^5^), 55.05 (s, C^17^), 45.97 (s, C^10^).

### 4.3. Synthesis of Ruthenium Complexes

#### 4.3.1. Preparation of [RuCl(η^6^-*p*-cymene)(**HL^1^**)] [CF_3_SO_3_], **1(TfO)**

AgCF_3_SO_3_ (0.043 g, 0.167 mmol) was added to a solution of [Ru(Cl)(μ-Cl)(*p*-cymene)]_2_ (0.056 g, 0.092 mmol) in acetonitrile (5 mL) and the mixture was stirred for 2 h at room temperature under argon. The resulting solution was filtered twice through Celite^TM^ 545 (Merck Millipore) to remove the silver chloride precipitate. The ligand **HL^1^** (0.059 g, 0.164 mmol) was added and the mixture was stirred for 24 h at room temperature under an inert atmosphere. The solvent was removed under vacuum and the residue was treated with ethyl ether (2 × 3 mL). The solid was filtered off and dried under vacuum over CaCl_2_/KOH. The presence of a mixture of the *E*/*Z* isomers was evidenced by analysis of the ^1^H NMR spectra (62:38 molar ratio, respectively). A solution of **1(TfO)** in CH_2_Cl_2_ and methanol (2:5) resulted in two types of single crystals. The X-ray diffraction analysis of these crystals showed the isolation of crystals of isomer *E* of **1(Cl)** and *Z* of **1(TfO)**.

Yield: 77 mg, 60%. Mp.: 180–181 °C. Anal. Calc. for C_29_H_35_ClF_3_N_3_O_6_RuS_2_ (779.25): C: 44.67, N: 5.39, H: 4.49, S: 8.21%; Exp. C: 44.51, N: 5.35, H: 4.28, S: 7.16%. MS(ESI) [*m*/*z* (%)]: 287.57 (35.4) |Ru(*p*-cymene)(**HL^1^**)|^2+^, 594.14(58) |Ru(*p*-cymene)(L^1^)|^+^. IR (ATR, cm^−1^): 3286 br (NH, OH), 1561 m (C=N), 818 m (C=S). 

**Scheme 6:**
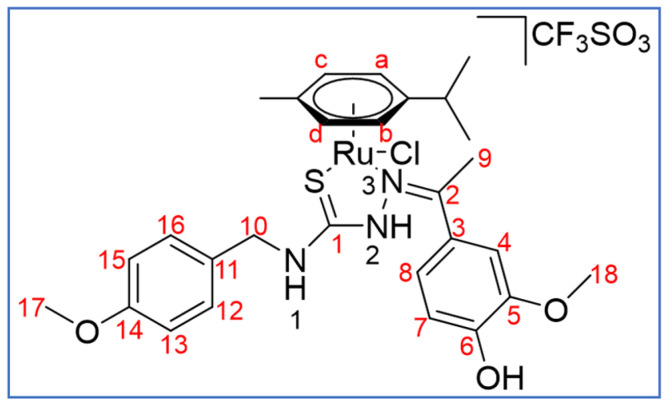
Atomic numbering scheme for **1(TfO)**.

^1^H NMR (400 MHz, CD_2_Cl_2_, ppm, see Scheme 6 for the atomic numbering used): *E* isomer: 10.84 (s, 1H, N^2^-H), 8.87 (b, 1H, N^1^-H), 7.84 (s, br, 1H, OH), 7.32 (d, 2H, ^3^*J*_H-H_ = 8.6 Hz, C^12^-H, C^16^-H), 7.18 (s, br, 1H, C^7^-H/C^8^-H), 7.07 (d, 1H, ^3^*J*_H-H_ = 8.1 Hz, C^7^-H/C^8^-H), 6.90 (d, 2H, ^3^*J*_H-H_ = 8.6 Hz, C^13^-H, C^15^-H), 6.13 (s, 1H, C^4^-H), 5.35, 4.91 (d, 2H, ^3^*J*_H-H_ = 5.9 Hz, C^a^-H, C^b^-H), 4.71, 4.01 (d, 2H, ^3^*J*_H-H_ = 5.9 Hz, C^c^-H, C^d^-H), 4.69 (m, 2H, C^10^-H), 4.06 (s, 3H, C^18^-H), 3.80 (s, 3H, C^17^-H), 2.74 (s, 3H, C^9^-H), 2.52 (br, 1H, ^3^*J*_H-H_ = 6.8 Hz, CH *^i^Pr*), 1.99 (s, 3H, CH_3_ *p*-cymene), 1.10, 1.02 (d, 6H, ^3^*J*_H-H_ = 6.8 Hz, CH_3_ *^i^Pr*); *Z* isomer: (400 MHz, CD_2_Cl_2_, ppm): 10.64 (s, 1H, N^2^-H), 8.42 (s, br, 1H, N^1^-H), 7.27 (d, 2H, ^3^*J*_H-H_ = 8.5 Hz, C^12^-H, C^16^-H), 6.87 (d, 2H, ^3^*J*_H-H_ = 8.7 Hz, C^13^-H, C^15^-H), 6.83 (d, 1H, ^3^*J*_H-H_ = 8.2 Hz, C^7^-H/C^8^-H), 6.75 (b, 1H, OH), 6.65 (d, 1H, ^3^*J*_H-H_ = 8.2 Hz, C^7^-H/C^8^-H), 5.67 (s, 1H, C^4^-H), 5.63, 5.47(d, 2H, ^3^*J*_H-H_ = 6.1 Hz, C^a^-H, C^b^-H,), 4.69–4.66 (d, 2H, ^3^*J*_H-H_ = 6.1 Hz, C^c^-H, C^d^-H), 4.64 (m, 2H, C^10^-H), 3.78 (s, 3H, C^17^-H), 3.68 (s, 3H, C^18^-H), 2.98 (s, 3H, C^9^-H), 2.76 (br, 1H, ^3^*J*_H-H_ = 7.2 Hz, CH *^i^Pr*), 2.26 (s, 3H, CH_3_ *p*-cymene), 1.23, 1.12 (d, 6H, ^3^*J*_H-H_ = 7.0 Hz, CH_3_ *^i^Pr*).

#### 4.3.2. Preparation of [RuCl(η^6^-*p*-cymene)(**HL^1^**)]Cl, **1(Cl)**

**HL^1^** (0.060 g, 0.167 mmol) was added to solution [Ru_2_(Cl)_2_(μ-Cl)_2_(η^6^-*p*-cymene)_2_] (0.051 g, 0.083 mmol) in methanol (5 mL) and the mixture was stirred for 4 h at room temperature under argon. To the resulting suspension was added NH_4_PF_6_ (0.027 g, 0.164 mmol), and the mixture was stirred for 24 h at room temperature under an inert atmosphere. The resulting solid was filtered off and dried under vacuum over CaCl_2_/KOH. ^1^H NMR spectroscopy led to the identification of the *E* isomer as the only species for the cationic complex [RuCl(η^6^-*p*-cymene)(HL^1^)]^+^ (**1^+^**). The ^31^P NMR spectrum did not contain any signals, and the elemental analysis data were consistent with the formation of compound **1(Cl)**.

Yield: 55mg, 43%. ^1^H NMR (400 MHz, CD_2_Cl_2_, ppm): 12.73 (s, 1H, N^2^-H), 10.89 (b, 1H, N^1^-H), 7.87 (s, br, 1H, OH), 7.33 (d, 2H, ^3^*J*_H-H_ = 8.6 Hz, C^12^-H, C^16^-H), 7.17 (s, br, 1H, C^7^-H/C^8^-H), 7.08 (d, 1H, ^3^*J*_H-H_ = 8.2 Hz, C^7^-H/C^8^-H), 6.91 (d, 2H, ^3^*J*_H-H_ = 8.7 Hz, C^13^-H, C^15^-H), 6.17 (s, 1H, C^4^-H), 5.35, 4.91 (d, 2H, ^3^*J*_H-H_ = 6.0 Hz, C^a^-H, C^b^-H), 4.71, 4.01 (d, 2H, ^3^*J*_H-H_ = 5.9 Hz, C^c^-H, C^d^-H), 4.69 (m, 2H, C^10^-H), 4.06 (s, 3H, C^18^-H), 3.80 (s, 3H, C^17^-H), 2.91 (s, 3H, C^9^-H), 2.55 (br, 1H, ^3^*J*_H-H_ = 6.8 Hz, CH *^i^Pr*), 1.99 (s, 3H, CH_3_ *p*-cymene), 1.11, 1.04 (d, 6H, ^3^*J*_H-H_ = 6.8 Hz, CH_3_ *^i^Pr*).

Single crystals suitable for X-ray diffraction were isolated from a CH_2_Cl_2_ solution (see Supplementary Materials).

#### 4.3.3. Preparation of [RuCl(η^6^-*p*-cymene) (**HL^2^**)][CF_3_SO_3_], **2(TfO)**

**2(TfO)** was obtained by following a procedure similar to that used for **1(TfO)**. The product was isolated as an orange solid. The presence of a mixture of the *E*/*Z* isomers was detected and determined by ^1^H NMR spectroscopy in a ~43:57 molar ratio, respectively. 

Yield: 107 mg, 81% Mp.: 163–165 °C. Anal. Calc. for C_27_H_30_ClF_4_N_3_O_5_RuS_2_ (753.19): C: 43.06, N: 5.58, H: 4.01, S: 8.51%; Exp. C: 43.15, N: 5.81, H: 3.90, S: 9.34%.

**Scheme 7:**
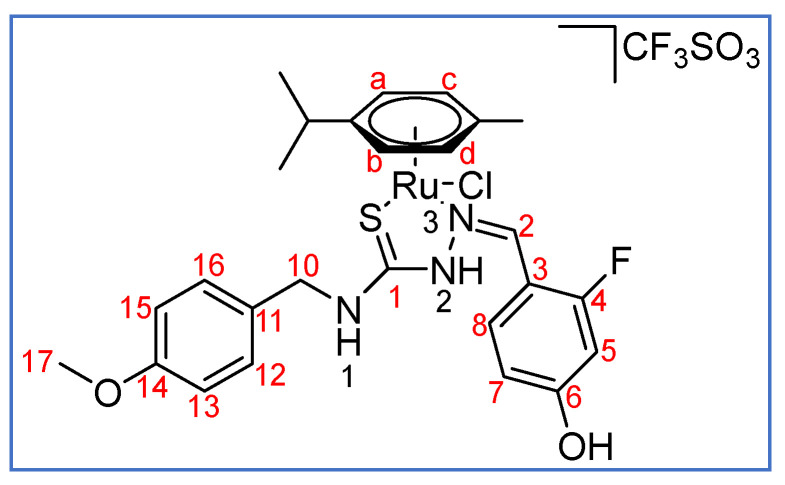
Atomic numbering scheme for **2(TfO)**.

MS(ESI) [*m*/*z* (%)]: 287.57 (35.4) |Ru(*p*-cymene)(HL^2^)|^2+^, 594.14(58) |Ru(*p*-cymene)(L^2^)|^+^. IR (ATR, cm^−1^): 3226 br (NH, OH), 1617 m (C=N), 816 m (C=S). 

^1^H NMR (400 MHz, CD_2_Cl_2_, ppm, see Scheme 7 for the atomic numbering used): *E* isomer, 10.62 (s, 1H, N^2^-H), 8.84 (s, br, 1H, N^1^-H), 8.65 (t, 1H, ^3^*J*_H-H_ = 8.6 Hz, C^8^-H), 8.55 (s, 1H, C^2^-H), 8.38 (s, br, 1H, OH), 7.27 (d, 2H, ^3^*J*_H-H_ = 8.7 Hz, C^12^-H, C^16^-H), 6.93 (dd, 1H, ^3^*J*_H-H_ = 8.2, ^4^*J*_H-H_ = 2.3 Hz, C^7^-H), 6.90 (t, 2H, ^3^*J*_H-H_ = 8.5 Hz, C^13^-H, C^15^-H), 6.81 (dd, 1H, ^3^*J*_H-F_ = 11.8, ^4^*J*_H-F_ = 2.2 Hz, C^5^-H), 5.50, 5.04 (d, 2H, C^a^-H, C^b^-H, ^3^*J*_H-H_ = 6.0 Hz), 4.93 (d, 2H, ^3^*J*_H-H_ = 6.0 Hz, C^c^-H, C^d^-H), 4.69 (m, 2H, C^10^-H), 3.83, (s, 3H, C^17^-H), 2.58 (br, 1H, ^3^*J*_H-H_ = 6.9 Hz, CH *^i^Pr*), 2.12 (s, 3H, CH_3_ *p*-cymene), 1.17, 1.09 (d, 3H, ^3^*J*_H-H_ = 6.9 Hz, CH_3_ *^i^Pr*); *Z* isomer: (400 MHz, CD_2_Cl_2_, ppm): 12.10 (s, 1H, N^2^-H), 8.58 (s, br, 1H, N^1^-H), 8.49 (s, 1H, C^2^-H), 8.20 (bs, 1H, OH), 7.27 (d, 2H, ^3^*J*_H-H_ = 8.7 Hz, C^12^-H, C^16^-H), 7.22 (t, 1H, ^3^*J*_H-H_ = 8.1Hz, C^8^-H), 6.90 (t, 2H, ^3^*J*_H-H_ = 8.5 Hz, C^13^-H, C^15^-H), 6.67 (dd, 1H, ^3^*J*_H-H_ = 8.2, ^4^*J*_H-H_ = 2.3 Hz, C^7^-H), 6.56 (dd, 1H, ^3^*J*_H-F_ = 11.8, ^4^*J*_H-H_ = 2.3 Hz, C^5^-H), 5.71, 5.57 (2H, ^3^*J*_H-H_ = 6.0 Hz, C^a^-H, C^b^-H), 5.60, 5.39 (d, 2H, ^3^*J*_H-H_ = 6.0 Hz, C^c^-H, C^d^-H), 4.68 (m, 2H, C^10^-H), 3.81 (s, 3H, C^17^-H), 2.79 (br, 1H, ^3^*J*_H-H_ = 6.9 Hz, CH *^i^Pr*), 2.69 (s, 3H, CH_3_ *p*-cymene) 1.29, 1.23 (d, 3H, ^3^*J*_H-H_ = 6.9 Hz, CH_3_ *^i^Pr*).

^19^F{^1^H} NMR (376 MHz, CD_2_Cl_2_, ppm): –70.13 (CF_3_SO_3_), –107.27 (***E*** isomer), −110.68 (***Z*** isomer).

A few single crystals from a solution of **2(TfO)** were obtained. X-ray analysis showed that these were [Ru_2_(L^2^)_2_(*p*-cymene)_2_][CF_3_SO_3_]_2_, **2′(TfO)**. Only sufficient crystals for characterization by MS and IR spectroscopies were obtained.

MS (ESI) [*m*/*z* (%)]: 594.14(58) |Ru(L^2^)(*p*-cymene)|^+^. IR (ATR, cm^−1^): 3271 br (NH, OH), 1617, 1557, 1511, 1459 m (C=N), 813 m (C=S).

#### 4.3.4. Preparation of [RuCl(η^6^-*p*-cymene)(**HL^2^**)]Cl, **2(Cl)**

To a suspension of [Ru(Cl)(μ-Cl)(*p*-cymene)]_2_ (0.05 g, 0.082 mmol) in methanol (5 mL) was added **HL^2^** (0.054 g, 0.164 mmol). The mixture was stirred for 4 h at room temperature under argon. To the resulting solution was added NH_4_PF_6_ (0.027 g, 0.164 mmol), and the mixture was stirred for 24 h at room temperature under an inert atmosphere. The precipitate was filtered off and the solvent was removed from the filtrate under vacuum. The resulting orange solid was dried under vacuum over CaCl_2_/KOH. ^1^H NMR spectroscopy showed that only the *E* isomer was obtained for the cation **2^+^**. The ^31^P NMR spectrum did not contain any signals, and the elemental analysis data were consistent with the formation of compound **2(Cl)**.

Yield: 79 mg, 76%. Anal. Calc. for C_26_H_30_Cl_2_FN_3_O_2_RuS (639.05): C: 48.82, N: 6.57, H: 4.73, S: 5.00%; Exp. C: 48.88, N: 6.29, H: 4.58, S: 4.94%. ^1^H NMR (CD_2_Cl_2_): 14.06 (s, 1H, N^2^-H), 9.49 (s, 1H, OH), 8.69 (s, 1H, N^1^-H), 8.44 (t, 1H, ^3^*J*_H-H_ = 8.7 Hz, C^8^-H), 8.38 (s, 1H, C^2^-H), 7.31 (d, 2H, ^3^*J*_H-H_ = 8.7 Hz, C^12^-H, C^16^-H), 6.97 (dd, 1H, ^3^*J*_H-H_ = 8.7 Hz, ^4^*J*_H-H_ = 2.3 Hz, C^7^-H), 6.93 (d, 2H, ^3^*J*_H-H_ = 8.7 Hz, C^13^-H, C^16^-H), 6.82 (dd, 1H, ^3^*J*_H-H_ = 11.9 Hz, ^4^*J*_H-H_ = 2.3 Hz, C^5^-H), 5.47, 4.99, 4.90 (d, 4H, ^3^*J*_H-H_ = 5.4 Hz, C^a^-H, C^b^-H, C^c^-H, C^d^-H), 4.71 (d, 2H, ^3^*J*_H-H_ = 5.7 Hz, C^10^-H), 3.81 (s, 3H, C^17^-H), 2.57 (br, 1H, ^3^*J*_H-H_ = 6.9 Hz, CH *^i^Pr*), 2.08 (s, 3H, CH_3_ *p*-cymene), 1.14, 1.07 (d, 6H, ^3^*J*_H-H_ = 7.0 Hz, CH_3_ *^i^Pr*).

### 4.4. Crystallography

The crystallographic data were collected at 100 K using a Bruker D8 Venture diffractometer with a Photon 100 CMOS detector and Mo-Kα radiation (λ = 0.71073 Å) generated by an Incoatec high brilliance microfocus source equipped with Incoatec Helios multilayer optics. APEX3 software was used to collect frames of data and index reflections and to determine the lattice parameters, SAINT was used for integration of the intensity of reflections, and SADABS was used for scaling and empirical absorption correction [37,38]. The structures were solved using the SHELXT program [39]. All non-hydrogen atoms were refined on F^2^ with anisotropic thermal parameters using SHELXL [40]. Hydrogen atoms were inserted at calculated positions and refined as riding atoms, except for those bonded to heteroatoms (N–H and O–H), which were generally located from the electron density synthesis Fo–Fc map and isotropically refined. Validation checking of the models (including for missed symmetry) was performed using PLATON [41], and plots of all structures were produced using MERCURY [42]. The crystallographic data collection and refinement parameters are listed in Table S1 (see Supplementary Materials).

The structure of the [Ru_2_(L^2^)_2_(*p*-cymene)_2_][O_3_SCF_3_]_2_ (**2′(TfO)**) showed the presence of a disordered triflate group. This disorder was modeled including two alternative positions for the anion with equivalent percentages of occupation.

### 4.5. Electrochemistry

Electrochemical measurements were carried out using an Autolab potentiostat/galvanostat (PGSTAT100). This system was equipped with a three-electrode cell, with a 2.00 mm diameter glassy carbon electrode as the working electrode (GCE), a platinum plate electrode as the counter electrode, and the Ag/AgCl reference electrode was used for electrochemical experiments.

The GCE was polished using alumina powder (0.03 µm) before use. Ferrocene was used as an internal reference, and the redox potentials presented in this work are related to the standard ferrocene/ferrocenium redox couple (Fc/Fc^+^).

Measurements were performed in 10^−3^ mol·L^−1^ solutions of compounds in methanol containing tetrabutylammonium perchlorate (TBAP) 0.1 mol·L^−1^ as supporting electrolyte. Solutions were deaerated by passing a stream of nitrogen through the solution for 10 minutes prior to measurement.

### 4.6. Cytotoxicity Assays

#### 4.6.1. Cell Line and Culture Conditions

Cytotoxicity studies on compounds were carried out on NCI-H460 (human lung carcinoma), A549 (human lung carcinoma), and MDA-MB231 (human breast adenocarcinoma) cell lines obtained from the American Type Culture Collection (ATCC). Cells were grown on culture media RPMI 1640 (NCI-H640) or DMEM (Dulbecco’s Modified Eagle’s Medium, A549 and MDA-MB231) supplemented with 10% FBS (Fetal Bovine Serum) and additionally with 2 mM L-glutamine for A549 and MDA-MB231, all under an atmosphere of 95% air and 5% CO_2_ at 37 °C.

#### 4.6.2. Cytotoxicity Study

Inhibition of cell growth induced by the test compounds was evaluated using a system based on the 3-[4,5-dimethylthiazol-2-yl]-2,-5-diphenyltetrazolium bromide assai (MTT) and on its ability to be transformed into formazan when the cells are metabolically active.

The cells were seeded in a sterile 96-well plate at a density of 5000–15,000 cells/well and incubated for 24 h in growth medium. A solution of the compound in DMSO was added to the cells while maintaining the same proportion of solvent in each well. After 72 h of incubation in an atmosphere of 95% air and 5% CO_2_ at 37 °C, 10 μL of 5 mg/mL MTT prepared in PBS (0.136 M NaCl, 1.47 mM KH_2_PO_4_, 8 mM NaH_2_PO_4_ and 2.68 mM KCl) was added to each well and the cell plate was incubated for another 4 h.

Subsequently, 10% SDS (100 μL) prepared in 0.01M HCl was added and the cell plate was incubated for 12–14 h under the same experimental conditions. Finally, the absorbance of the samples on the cell plate was measured at a wavelength of 595 nm (Tecan M1000 infinite Pro, Tecan, Hombrechtikon, Switzerland). All experiments were carried out in triplicate. The absorbance measurement range was assessed between one value (average of triplicate points) containing 5000–15,000 cells in RPMI 1640/DMEM media in the absence of growth factors (which allows the stable cell concentration to be determined) and another value (average of triplicate points) containing the usual growth medium, allowing the maximum cell growth at 48–72 h to be determined.

Controls with DMSO at the same proportion in which the compounds were dissolved were included in all experiments. These controls showed a cell growth inhibition of 6–8% with respect to the control, in which the cells were grown in the growth medium.

### 4.7. Analysis and Expression of the Results

The experiments were carried out in triplicate. Data are expressed as % growth inhibition, calculated using the following formula:

(1) $$\%\;inhibition=100-\frac{\left(AO\times 100\right)}{AT}$$

where *AO* is the absorbance observed in the wells with the compounds under study and *AT* is the absorbance observed in the wells with DMSO controls. 

When concentration-dependent cell growth inhibition was observed, the inhibitory potency was evaluated by calculating the concentration–percent inhibition curve of the compound by adjustment to the following equation:

(2) $$y=\frac{E_{max}}{1+{\left(\frac{IC_{50}}{x}\right)}^{n}}$$

## Figures and Tables

**Table 1:** Relevant ^1^H NMR chemical shift values (δ in ppm) for **HL^1^** and **HL^2^** and their complexes in CD_2_Cl_2_.

		N^2^–*H*	N^1^–*H*	N^3^=C^2^*H*	N^3^=CC^9^*H*_3_	*Ha*,*bHc*,*d*	–CH_3_ *p*-cym	–^i^Pr *p*-cym
**HL^1^**		8.66	7.85		2.25			
**1(Cl)**	*E* ^a^	12.73	10.89		2.91	5.35; 4.91; 4.71; 4.01	1.99	1.11; 1.04
**1(TfO)**	*E* ^a^	10.84	8.87		2.74	5.35; 4.91; 4.71; 4.01	1.99	1.10; 1.02
	*Z* ^a^	10.64	8.42		2.98	5.63; 5.47; 4.66; 4.60	2.26	1.23; 1.12
**HL^2^**		8.97	7.68	7.91				
**2(Cl)**	*E* ^a^	14.06	8.69	8.38		5.47; 4.99; 4.90	2.08	1.14; 1.07
**2(TfO)**	*E* ^a^	10.62	8.84	8.55		5.50; 5.04; 4.93	2.12	1.17; 1.09
	*Z* ^a^	12.10	8.58	8.49		5.71, 5.57; 5.60, 5.39	2.69	1.29; 1.23

^a^ Signals corresponding to the isomer related to the conformation of the C2=N3 bond.

**Table 2:** Selected bond lengths (Å) and angles (°).

	HL^1^	1(Cl).MeOH	1(TfO).2(MeOH)		HL^2^	2′(TfO)
			A	B		
X =		Cl(1)	Cl(1)	Cl(2)		S(1) ^d^
Ru(1)–N(3)		2.1638(13)	2.127(4)	2.126(4)		2.104(3)
Ru(1)–S(1)		2.3464(4)	2.3744(13)	2.3767(13)		2.3617(10)
Ru(1)–X		2.4238(4)	2.3997(14)	2.4015(14)		2.4085(9)
Ru(1)–Cc ^b^		1.6913(2)	1.6965(4)	1.6907(4)		1.4728(3)
Ru(1)–Cm ^c^		2.208(1)	2.208(2)	2.206(2)		2.215(2)
S(1)–C(1)	1.6897(13)	1.6970(16)	1.698(5)	1.701(5)	1.699(3) ^a^	1.795(3)
N(2)–C(1)	1.3628(16)	1.349(2)	1.348(6)	1.333(6)	1.356(4) ^a^	1.303(4)
N(2)–N(3)	1.3934(14)	1.3960(18)	1.387(6)	1.413(6)	1.370(4) ^a^	1.397(4)
N(3)–C(2)	1.2909(16)	1.303(2)	1.304(6)	1.293(6)	1.279(4) ^a^	1.295(4)
C(1)–N(1)	1.3326(16)	1.332(2)	1.329(7)	1.339(7)	1.327(4) ^a^	1.340(5)
N(1)–C(11)	1.4520(16)	1.454(2)	1.478(7)	1.468(7)	1.465(4) ^a^	1.461(5)
N(3)–Ru(1)–S(1)		82.30(4)	80.99(12)	81.09(12)		79.10(9)
N(3)–Ru(1)–X		87.06(4)	84.63(12)	84.65(12)		83.43(9)
S(1)–Ru(1)–X		87.307(15)	88.75(5)	88.80(5)		81.75(3)
S(1)–Ru(1)–Cc ^b^		125.504(12)	126.86(4)	127.03(4)		131.25(3)
N(3)–Ru(1)–Cc ^b^		132.05(4)	132.57(11)	132.60(12)		129.51(9)
X–Ru(1)–Cc ^b^		127,322(11)	127.24(4)	126.95(4)		132.48(3)
C(2)–N(3)–Ru(1)		129.91(11)	130.7(4)	132.1(4)		121.4(3)
N(2)–N(3)–Ru(1)		114.87(9)	114.4(3)	114.1(3)		120.9(2)
C(1)–S(1)–Ru(1)		99.90(6)	98.77(18)	98.72(18)		95.04(13)
C(2)–N(3)–N(2)	116.67(11)	114.81(13)	120.8(4)	120.4(4)	118.5(3) ^a^	117.4(3)
N(3)–C(2)–C(3)	115.67(11)	119.63(14)	122.5(5)	123.3(5)	120.0(4) ^a^	131.6(4)
N(1)–C(1)–N(2)	116.15(11)	116.04(14)	115.8(5)	116.1(5)	115.5(3) ^a^	121.6(3)
N(1)–C(1)–S(1)	124.78(10)	121.15(12)	124.3(4)	123.4(4)	124.3(2) ^a^	114.8(3)
N(2)–C(1)–S(1)	119.06(9)	122.81(13)	119.9(4)	120.5(4)	120.1(2) ^a^	123.5(3)

^a^ Data are the average of those observed in the two molecules present in the asymmetric unit. ^b^ Centroid defined from the averages of the six-membered ring of the *p*-cymene ligand. ^c^ Average distance of the six Ru–C distances of the *p*-cymene ligand. ^d^ Symmetry transformations used to generate equivalent atoms: −x + 1, −y + 1, −z.

**Table 3:** Electrochemical data for complexes and the corresponding ligand in methanol at a scan rate of 0.2 V/s.

Compound	E_pa_ (V)	E_pc_ (V)	E_pa_ − E_pc_
**HL^1^**	0.59	-	
	−0.61	−0.75	0.14
**1(TfO)**	0.46	0.07	
	0.63	-	
	−0.59	−0.71	0.12
**HL^2^**	0.58	-	
	−0.40	−0.80	
**2(TfO)**	0.49	-	
	0.72	-	
	−0.57	−0.73	0.16

**Table 4:** Summary of the results of the cytotoxicity screening of the compounds.

	NCl-H460 ^#1^			A549 ^#2^			MDA-MB-231 ^#3^		
	Inh ^#4^	IC_50_	E_max_^#5^	Inh ^#4^	IC_50_	E_max_^#5^	Inh ^#4^	IC_50_	E_max_^#5^
**HL^1^**	19(3)				29.6(2.12)	62(1)		34.2(0.9)	59(3)
**1(TfO)**	41(4)				28.5(0.48)	92(2)		30.7(0.9)	89(1)
**HL^2^**	49(1)			50(3)			37(2)		
**2(TfO)**	40(2)				52.6(17.1)	55(2)	49(1)		
**Cisplatin**		6.1(0.3)	68(2)		10.0(0.28)	84(1)		17.9(0.7)	79(1)

^#1^ Non-small-cell lung carcinoma cell line; ^#2^ Non-small-cell lung carcinoma estrogen receptor positive; ^#3^ Tumor cells of triple negative (estrogen and progesterone receptors and HER2 amplification gen) breast adenocarcinoma; ^#4^ % Inhibition growth at a concentration of 100 μM; **^#5^** Efficacy of inhibition expressed at maximum % inhibition of cell growth.

## Data Availability

CCDC 2216181-2216186 contain the supplementary crystallographic data for this paper. These data can be obtained free of charge via www.ccdc.cam.ac.uk/data_request/cif (accessed on 30 October 2022), by emailing data_request@ccdc.cam.ac.uk, or by contacting The Cambridge Crystallographic Data Centre, 12 Union Road, Cambridge CB2 1EZ, UK; fax: +44 1223 336033.

## Supplementary Materials

The following supporting information can be downloaded at: https://www.mdpi.com/article/10.3390/molecules27227976/s1, Tables S1 and S2: Crystal data and structure refinement, Selected bond lengths and angles, and Main intermolecular interactions. Figures S1–S4: 1H NMR spectra of the ligands and their complexes. Figures S5 and S6: 13C NMR spectra of the ligands. Figures S7 and S8: NOESY spectra of the complexes. Figures S9–S12: ESI mass spectra of the ligands and complexes. Figures S13–S16: IR (solid) of the ligands and complexes. Figure S17–S19: cyclic voltammogram studies.
