# Supplementary material for: Synthesis, Characterization, and Cytotoxicity Studies of N-(4-Methoxybenzyl) Thiosemicarbazone Derivatives and Their Ruthenium(II)-p-cymene Complexes
Source: Molecules. 2022 Nov 17;27(22):7976. doi: 10.3390/molecules27227976 (PMC9696800; doi:10.3390/molecules27227976)
Supplement: Supplementary file 1 [file molecules-27-07976-s001.zip › molecules-2034757-supplementary.pdf]

Synthesis, Characterization, and Cytotoxic Studies of N-(4-methoxybenzyl) Thiosemicarbazone Derivatives and Their Ruthenium(II)-p-cymene complexes

Mónica Martínez-Estévez[a], Soledad García-Fontán[a],[b],*, Saray Argibay-Otero[a],[b], Inmaculada Prieto[b],[c], Ezequiel M. Vázquez-López [a],[b],*

[a] Departamento de Química Inorgánica, Universidade de Vigo, Campus Universitario, E-36310 Vigo, Galicia – Spain.

[b] Metallosupramolecular Chemistry Group Galicia South Health Research Institute (IIS Galicia Sur) SERGAS-UVIGO. Galicia – Spain.

[c] Departamento de Química Física, Universidade de Vigo, Campus Universitario, E-36310 Vigo, Galicia – Spain.

* Corresponding authors: S.G.F, e-mail: sgarcia@uvigo.gal

E.M.V.L, e-mail: ezequiel@uvigo.gal

Supplementary Material

Contents:

Crystal data 3

Table S1. Crystal data and structure refinement. 3

Table 2. Selected bond lengths (Å) and angles (°) 5

Table S3. Main intermolecular interactions observed in the crystal structures (Å and °). 7

^{1}H-NMR spectra 9

Figure S1: ^{1}H NMR spectrum of ligand HL^1 9

Figure S2: ^{1}H NMR spectrum of ligand HL^2 9

Figure S3: ^{1}H NMR spectrum of complex 1(TfO) 10

Figure S4: ^{1}H NMR spectrum of complex 2(TfO) 10

^{13}C-NMR spectra 11

Figure S5: ^{13}C NMR spectrum of ligand HL^1 11

Figure S6: ^{13}C NMR spectrum of ligand HL^2 11

NOESY spectra 12

Figure S7: NOESY spectrum of complex 1(OTf) 12

Figure S8: NOESY spectrum of complex 2(OTf) 12

ESI-Mass spectra 13

Figure S9: ESI Mass spectra of ligand HL^1 13

Figure S10: ESI Mass spectra of ligand HL^2 13

Figure S11: ESI Mass spectra of complex 1(TfO) 14

Figure S12: ESI Mass spectra of complex 2(TfO) 14

Infrared spectra 15

Figure S13: IR spectrum of ligand HL^1 15

Figure S14: IR spectrum of ligand HL^2 15

Figure S15: IR spectrum of complex 1(TfO) 15

Figure S16: IR spectrum of complex 2(TfO) 15

Cyclic voltammetry 16

Figure S17: Cyclic voltammetry (CV) and square wave voltammetry (SWV) data of complex 1(TfO) 16

Figure S18: Cyclic voltammetry (CV) and square wave voltammetry (SWV) data of complex 2(TfO) 16

Figure S19: X: I_{pa} (●) and I_{pc} (▪) of redox waves for complexes 1(TfO) (top) and 2(TfO) (bottom) vs square root of scan rate. 17

Crystal data

Table S1. Crystal data and structure refinement.

Compound	HL^1	HL^2	**1(Cl)**.(CH_3OH)
CSD deposition number	2216181	2216182	2216183
Empirical formula	$C_{18}H_{20}N_3O_3S$	$C_{16}H_{16}FN_3O_2S$	$C_{29}H_{39}Cl_2N_3O_4RuS$
Formula weight	358.43	333.38	697.66
Temperature (K)	100(2)	100(2)	100(2)
Wavelength (Å)	0.71073	0.71073	0.71073
Crystal system	Monoclinic	Triclinic	Monoclinic
Space group	P *2₁/c*	P *-1*	P *2₁/n*
Unit cell dimensions			
a(Å)	5.1245(2)	9.298(3)	13.1447(9)
b(Å)	34.1354(13)	9.575(3)	10.2221(6)
c(Å)	9.8803(4)	17.659(5)	22.8513(16)
α(°)	90	96.942(10)	90
β(°)	95.0150(10)	90.667(10)	99.019(2)
γ(°)	90	97.968(10)	90
Volume ($Å^3$)	1721.71(12)	1544.9(8)	3032.5(3)
Z	4	4	4
ρ_c (Mg/m^3)	1.383	1.433	1.528
μ(mm^{-1})	0.211	0.233	0.801
θ range (°)	2.39 - 28.30°.	2.16 to 25.89	1.92 - 28.32
Index ranges (h;k;l)	-6,6; -45,45; -11,13	11,11; -11,11; -21,21	-17,17; -13,13; -30,30
Reflections collected	39775	43317	55640
Independent reflections (R_{int})	4265 (0.0196)	5754 (0.1189)	7560 (0.0364)
Goodness-of-fit on F^2	1.049	1.155	1.047
Final R1/wR2 [I>2σ(I)]	0.0396/0.1097	0.0859/0.1686	0.0252/0.0624
R1/wR2 (all data)	0.0406/0.1107	0.11870.1854	0.0292/0.0645
Diff. peak and hole (e $Å^3$)	0.873, -0.766	0.660, -0.432	0.906/-0.654

Table S1. (cont.)

Compound	**1(Cl)**.$^{4}/_{3}H_2O$	**1(TfO)**.2(CH_3OH)	**2'(TfO)**
CSD deposition number	2216184	2216185	2216186
Empirical formula	$C_{28}H_{38}Cl_2N_3O_{4.33}RuS$	$C_{31}H_{43}ClF_3N_3O_8RuS_2$	$C_{54}H_{58}F_8N_6O_{10}Ru_2S_4$
Formula weight	6100(2)89.98	843.32	1433,44
Temperature (K)	100(2)	100(2)	292(2)
Wavelength	0.71073	0.71073 Å	0.71073 Å
Crystal system	Trigonal	Triclinic	Triclinic
Space group	R-3	P *-1*	P *-1*
a(Å)	40.755(2)	12.4823(9)	9.9016(3)
b(Å)	40.755(2)	16.6642(12)	12.4709(4)
c(Å)	10.0513(8)	18.6059(14)	13.2967(4)
α(°)	90	92.560(3)	107.2590(10)
β(°)	90	90.174(3)	91.1350(10)
γ(°)	120	111.434(2)	109.7010(10)
Volume (Å^3)	14458.2(18)	3598.1(5)	1463.02(8)
Z	18	4	1
ρ_c (Mg/m^3)	1.426	1.557	1.627
μ(mm^{-1})	0.756	0.694	0.746
θ range (°)	2.332 - 28.298	2.04 to 28.39	1.83 to 28.31
Index ranges (h;k;l)	-54,52; -42,54, -13,13	-16,16; -22,22; -23,24	-13,13; -16,16; -17;17
Reflections collected	83112	72492	73493
Independent reflections (R_{int})	7989 (0.0680)	17900 (0.0365)	7278 (0.0328)
Goodness-of-fit on F^2	1.051	1.226	1.050
Final R1/wR2 [I>2σ(I)]	0.0393/0.0888	0.0781/0.1667	0.0536/0.1389
R1/wR2 (all data)	0.0586/0.0996	0.0837/0.1690	0.0609/0.1460
Diff. peak and hole (e Å^3)	0.794/-0.764	3.497/-2.428	0.983/-1.261

Table 2. Selected bond lengths (Å) and angles (°)

	**HL**[1]	**1(Cl)**.CH_3OH	**1(Cl)**.$^4/_3H_2O$	**1(TfO)**.2(CH_3OH)	
				A	B
X =		Cl(1)	Cl(1)	Cl(1)	Cl(2)
Ru(1)-N(3)		2.1638(13)	2.139(2)	2.127(4)	2.126(4)
Ru(1)-S(1)		2.3464(4)	2.3422(8)	2.3744(13)	2.3767(13)
Ru(1)-X		2.4238(4)	2.4278(7)	2.3997(14)	2.4015(14)
Ru(1)-Cc[b]		1.6913(2)	1.7200(2)	1.6965(4)	1.6907(4)
Ru(1)-Cm[c]		2.208(1)	2.198(1)	2.208(2)	2.206(2)
S(1)-C(1)	1.6897(13)	1.6970(16)	1.695(3)	1.698(5)	1.701(5)
N(2)-C(1)	1.3628(16)	1.349(2)	1.339(4)	1.348(6)	1.333(6)
N(2)-N(3)	1.3934(14)	1.3960(18)	1.404(3)	1.387(6)	1.413(6)
N(3)-C(2)	1.2909(16)	1.303(2)	1.293(4)	1.304(6)	1.293(6)
C(1)-N(1)	1.3326(16)	1.332(2)	1.334(4)	1.329(7)	1.339(7)
N(1)-C(11)	1.4520(16)	1.454(2)	1.451(4)	1.478(7)	1.468(7)
N(3)-Ru(1)-S(1)		82.30(4)	82.45(6)	80.99(12)	81.09(12)
N(3)-Ru(1)-X		87.06(4)	86.54(6)	84.63(12)	84.65(12)
S(1)-Ru(1)-X		87.307(15)	87.13(3)	88.75(5)	88.80(5)
S(1)-Ru(1)-Cc[b]		125.504(12)	124.98(2)	126.86(4)	127.03(4)
N(3)-Ru(1)-Cc[b]		132.05(4)	133.64(7)	132.57(11)	132.60(12)
X-Ru(1)-Cc[b]		127,322(11)	126.61(2)	127.24(4)	126.95(4)
C(2)-N(3)-Ru(1)		129.91(11)	130.6(2)	130.7(4)	132.1(4)
N(2)-N(3)-Ru(1)		114.87(9)	114.95(18)	114.4(3)	114.1(3)
C(1)-S(1)-Ru(1)		99.90(6)	99.44(10)	98.77(18)	98.72(18)
C(2)-N(3)-N(2)	116.67(11)	114.81(13)	114.1(2)	120.8(4)	120.4(4)
N(3)-C(2)-C(3)	115.67(11)	119.63(14)	120.7(3)	122.5(5)	123.3(5)
N(1)-C(1)-N(2)	116.15(11)	116.04(14)	116.1(3)	115.8(5)	116.1(5)
N(1)-C(1)-S(1)	124.78(10)	121.15(12)	122.5(3)	124.3(4)	123.4(4)
N(2)-C(1)-S(1)	119.06(9)	122.81(13)	121.4(2)	119.9(4)	120.5(4)

a) The data are the average of those observed in the two molecules present in the asymmetric unit.
b) Centroid defined for the averages of the six-membered ring of p-cymene ligand.
c) Average distance of the six Ru-C of p-cymene ligand.
d) Symmetry transformations used to generate equivalent atoms: -x+1,-y+1,-z

Table 2. (cont.)

	HL^2	2'(TfO)
X =		S(1)[d]
Ru(1)-N(3)		2.104(3)
Ru(1)-S(1)		2.3617(10)
Ru(1)-X		2.4085(9)
Ru(1)-Cc[b]		1.4728(3)
Ru(1)-Cm[c]		2.215(2)
S(1)-C(1)	1.699(3) [a]	1.795(3)
N(2)-C(1)	1.356(4) [a]	1.303(4)
N(2)-N(3)	1.370(4) [a]	1.397(4)
N(3)-C(2)	1.279(4) [a]	1.295(4)
C(1)-N(1)	1.327(4) [a]	1.340(5)
N(1)-C(11)	1.465(4) [a]	1.461(5)
N(3)-Ru(1)-S(1)		79.10(9)
N(3)-Ru(1)-X		83.43(9)
S(1)-Ru(1)-X		81.75(3)
S(1)-Ru(1)-Cc[b]		131.25(3)
N(3)-Ru(1)-Cc[b]		129.51(9)
X-Ru(1)-Cc[b]		132.48(3)
C(2)-N(3)-Ru(1)		121.4(3)
N(2)-N(3)-Ru(1)		120.9(2)
C(1)-S(1)-Ru(1)		95.04(13)
C(2)-N(3)-N(2)	118.5(3) [a]	117.4(3)
N(3)-C(2)-C(3)	120.0(4) [a]	131.6(4)
N(1)-C(1)-N(2)	115.5(3) [a]	121.6(3)
N(1)-C(1)-S(1)	124.3(2) [a]	114.8(3)
N(2)-C(1)-S(1)	120.1(2) [a]	123.5(3)

a) The data are the average of those observed in the two molecules present in the asymmetric unit.
b) Centroid defined for the averages of the six-membered ring of p-cymene ligand.
c) Average distance of the six Ru-C of p-cymene ligand.
d) Symmetry transformations used to generate equivalent atoms: -x+1,-y+1,-z

Table S3. Main intermolecular interactions observed in the crystal structures (Å and °).				
D-H...A	d(D-H)	d(H...A)	d(D...A)	<(DHA)
**HL**[1]				
O(2)-H(2)...O(1)[#1]	0.80(3)	2.12(3)	2.7946(15)	141(2)
O(2)-H(2)...O(3)	0.80(3)	2.25(2)	2.6960(14)	116(2)
N(2)-H(1N2)...S(1)[#2]	0.89(2)	2.60(2)	3.4104(12)	151.2(17)
**HL**[2]				
N(1A)-H(1A)...N(3A)	0.85(5)	2.11(5)	2.557(5)	112(4)
N(2A)-H(2A1)...S(1B)[#4]	0.83(5)	2.68(5)	3.456(4)	155(5)
O(2A)-H(2A2)...S(1A)[#3]	0.81(5)	2.48(5)	3.285(4)	175(4)
N(1B)-H(1B)...N(3B)	0.88(4)	2.15(4)	2.586(5)	110(3)
N(2B)-H(2B1)...S(1A)[#4]	0.72(5)	2.77(5)	3.432(4)	155(5)
O(2B)-H(2B2)...S(1B)[#3]	0.82(6)	2.45(6)	3.261(4)	173(5)
**1(Cl).CH_3OH**				
N(1)-H(1)...Cl(2)	0.77(2)	2.40(2)	3.1398(15)	160(2)
N(2)-H(2N2)...Cl(2)	0.84(2)	2.47(2)	3.2317(15)	152(2)
O(2)-H(2)...O(1S)	0.81(3)	1.86(3)	2.654(2)	171(3)
O(1S)-H(1S)...Cl(2)[#5]	0.83(3)	2.25(3)	3.0756(16)	174(3)
**1(Cl)**.$^{4}/_{3}H_2O$				
N(1)-H(1)...Cl(2)	0.85(4)	2.32(4)	3.116(4)	158(3)
C(4)-H(4)...Cl(2)[#6]	0.95	2.83	3.635(3)	143.0
O(2)-H(6)...Cl(1)[#7]	0.93(4)	2.45(4)	3.197(2)	138(3)
O(2)-H(6)...O(3)	0.93(4)	2.17(4)	2.683(3)	114(3)
N(2)-H(9)...Cl(2)	0.82(4)	2.43(4)	3.186(3)	153(3)
C(11)-H(11D)...S(1)	0.99	2.54	3.066(4)	113.1
C(14)-H(14)...Cl(1)[#8]	0.95	2.91	3.783(3)	153.2
O(1W)-H(11W)...O(1W)[#6]	1.000(2)	2.55(10)	3.138(11)	117(8)
O(1W)-H(12W)...Cl(2)	1.000(2)	2.51(5)	3.238(9)	129(5)
**1(TfO).2(CH_3OH)**				
N(1A)-H(1A)...O(2A)[#9]	0.80(7)	2.50(7)	3.071(6)	129(6)
N(1A)-H(1A)...O(3A)[#9]	0.80(7)	2.25(7)	3.031(6)	165(7)
N(2A)-H(2A)...O(2A)[#9]	0.88	2.00	2.816(6)	152.8
O(2A)-H(2A1)...O(2M)	0.73(7)	1.85(7)	2.570(6)	171(8)
N(1B)-H(1B)...O(2B)[#10]	0.79(6)	2.38(6)	3.047(6)	143(5)
N(1B)-H(1B)...O(3B)[#110]	0.79(6)	2.39(6)	3.103(6)	150(5)
N(2B)-H(2B)...O(2B)[#9]	0.88	2.04	2.858(6)	153.5
O(2B)-H(2B1)...O(1M)	0.72(6)	1.86(6)	2.577(7)	169(7)
O(1M)-H(1M)...S(1T)	0.84	3.02	3.824(5)	161.3
O(1M)-H(1M)...O(1T3)	0.84	1.87	2.706(7)	174.4
O(2M)-H(2M)...O(2T3)	0.77(9)	1.93(9)	2.696(7)	173(10)
O(3M)-H(3M)...O(4M)	0.84	2.14	2.882(14)	147.7
O(4M)-H(4M)...O(2T2)	0.857(19)	2.20(9)	2.879(10)	136(11)

Table S3. (cont.)				
**[Ru_2(p-Cm)$_2$(μ-L^2)$_2$][SO_3CF_3]**				
N(1)-H(1A)...O(1T3^a)#11	0.86	2.07	2.839(11)	148.1
N(1)-H(1A)...O(2T3^b)#11	0.86	2.30	3.02(2)	141.5
O(2)-H(2B)...O(1T2^a)	0.82	2.09	2.868(12)	158.3
O(2)-H(2B)...O(2T1^b)	0.82	1.88	2.648(9)	156.0
Symmetry transformations used to generate equivalent atoms: #1 x+1,-y+3/2, z-1/2; #2 -x+1,-y+1,-z; #3 x-1,y-1,z; #4 -x+1,-y+1,-z+1; #5 -x+1,-y+1,-z; #6 x-y,x-1,-z+1; #7 x-y,x-1,-z; #8 x,y,z+1; #9 -x+1,-y+1,-z; #10 -x+2,-y+1,-z+1; #11 x-1,y-1,z-1				

^{1}H-NMR spectra

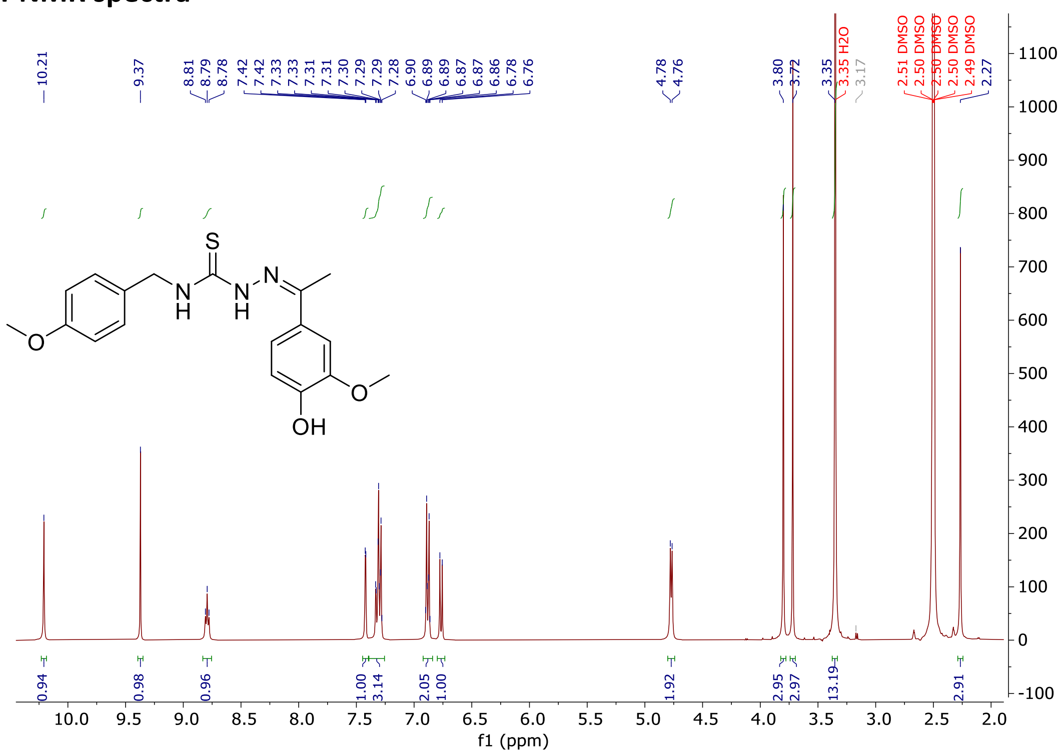

Figure S1: ^{1}H NMR spectrum of ligand HL[1]

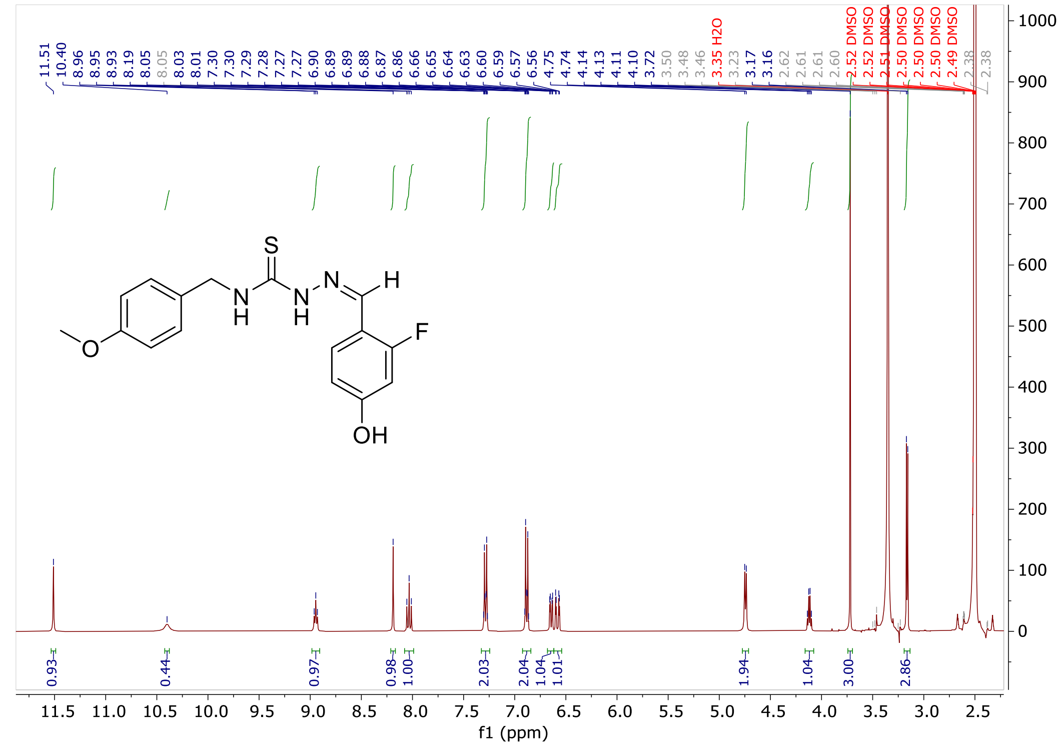

Figure S2: ^{1}H NMR spectrum of ligand HL[2]

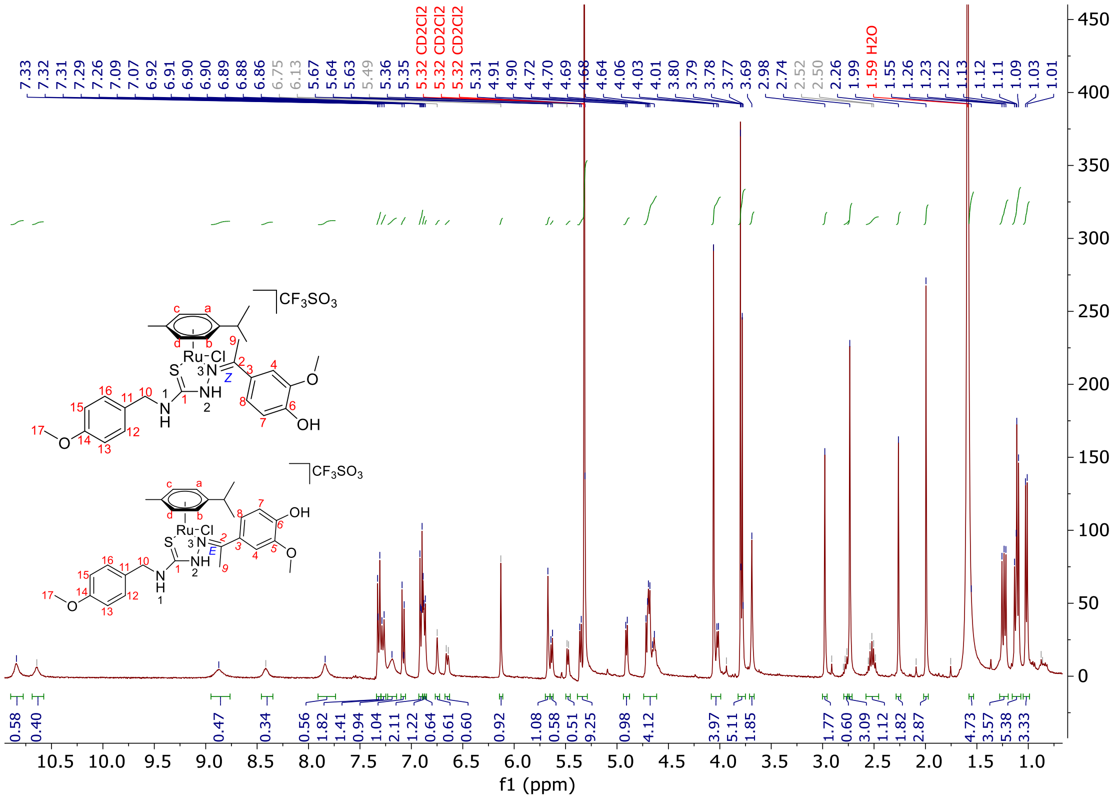

Figure S3: ^{1}H NMR spectrum of complex 1(TfO)

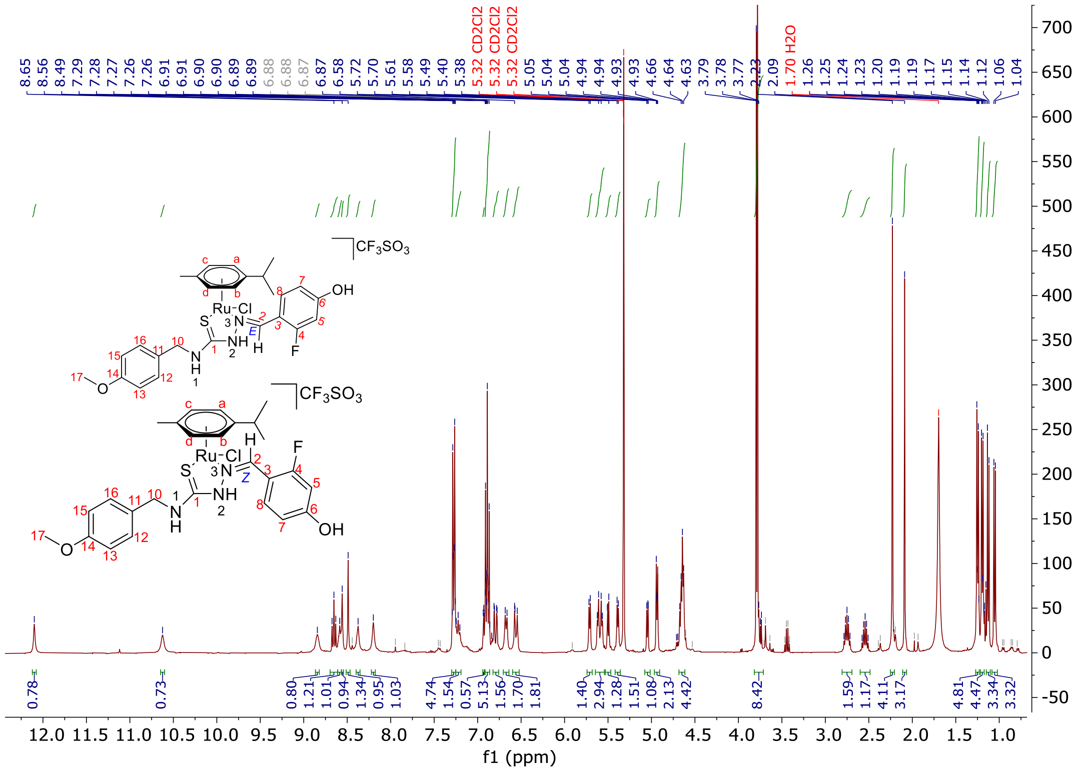

Figure S4: ^{1}H NMR spectrum of complex 2(TfO)

^{13}C-NMR spectra

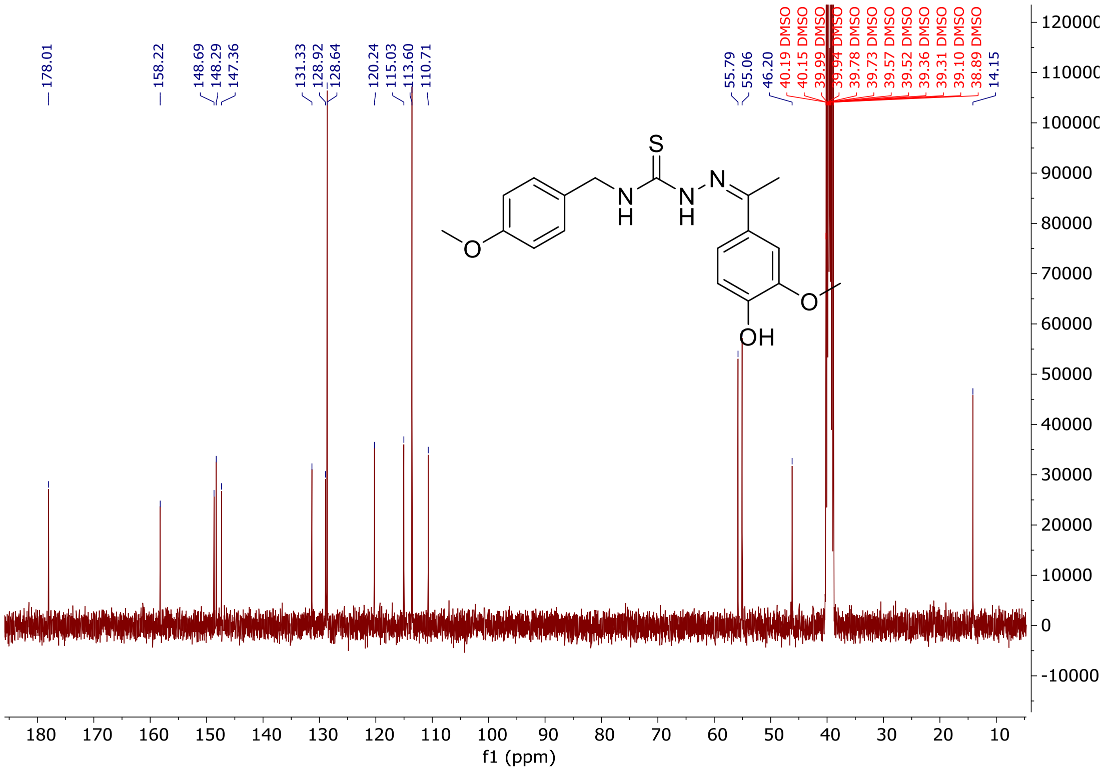

Figure S5: ^{13}C NMR spectrum of ligand HL^1

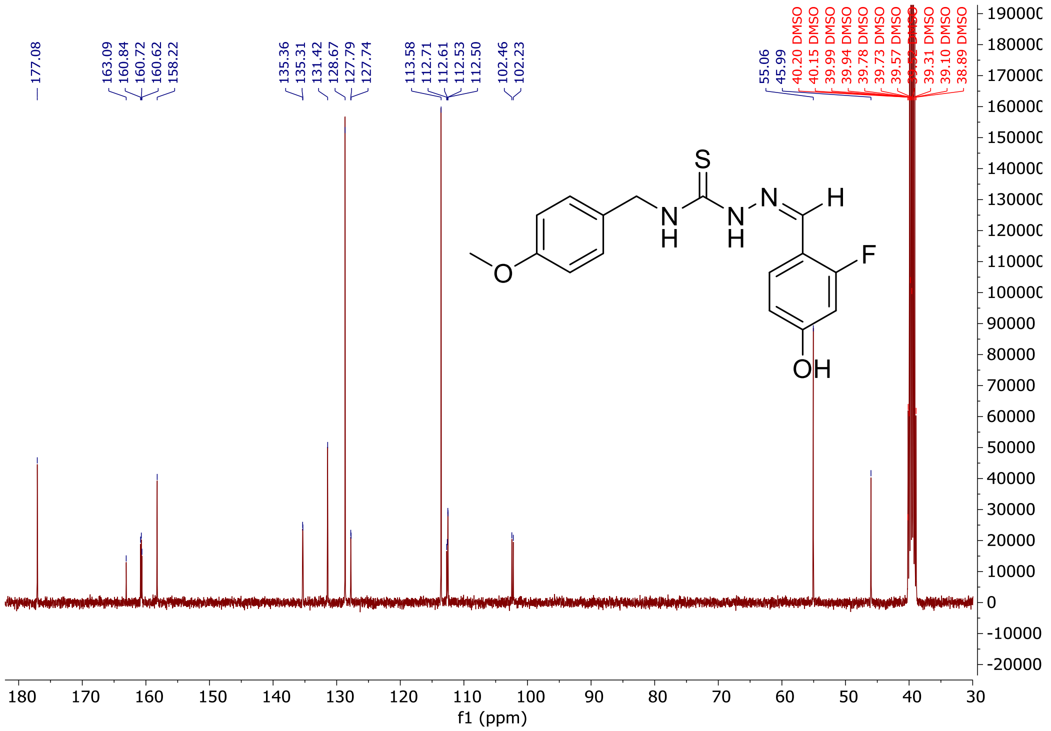

Figure S6: ^{13}C NMR spectrum of ligand HL^2

NOESY spectra

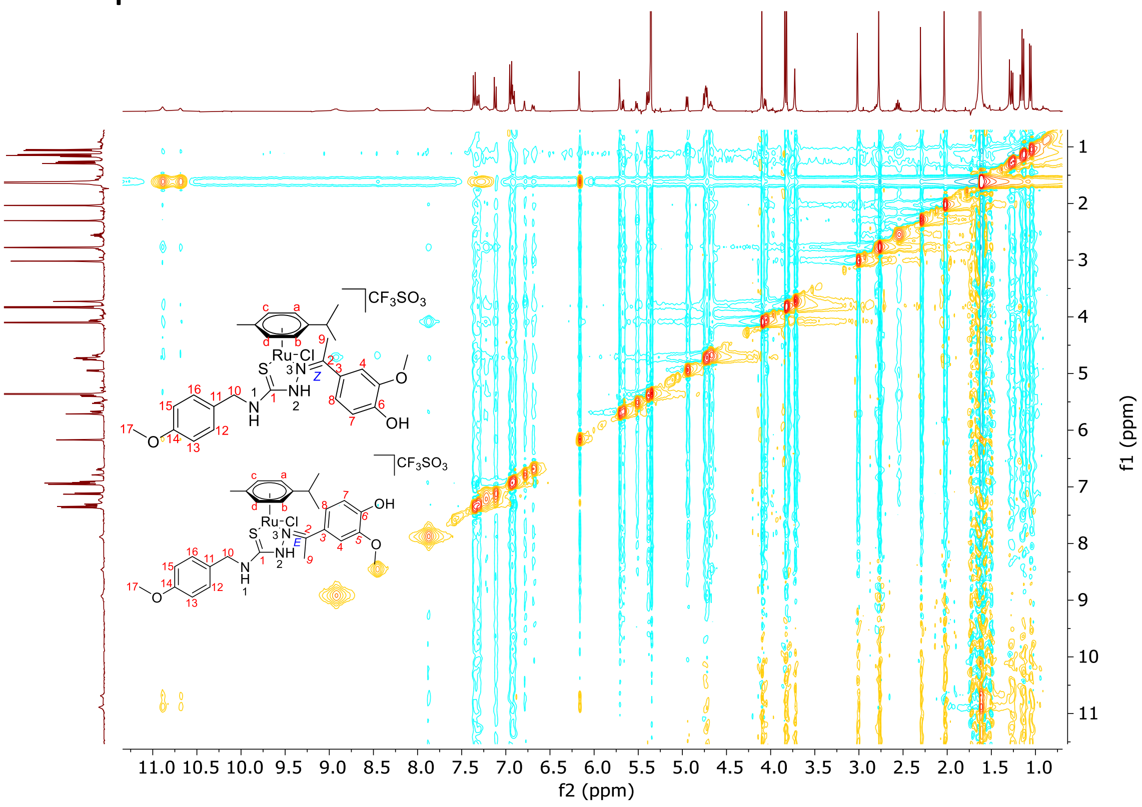

Figure S7: NOESY spectrum of complex 1(OTf)

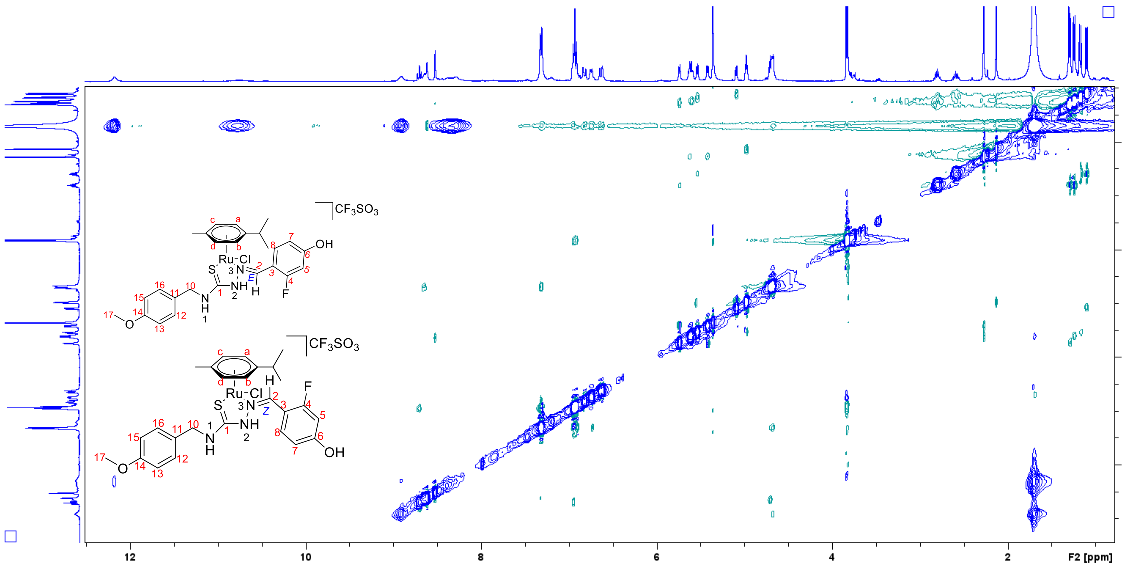

Figure S8: NOESY spectrum of complex 2(OTf)

ESI-Mass spectra

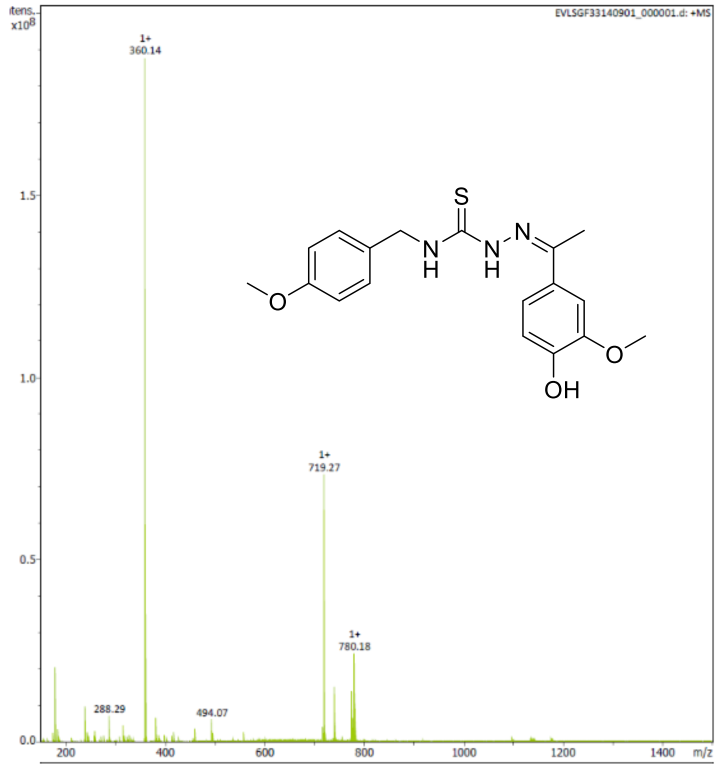

Figure S9: ESI Mass spectra of ligand HL^1

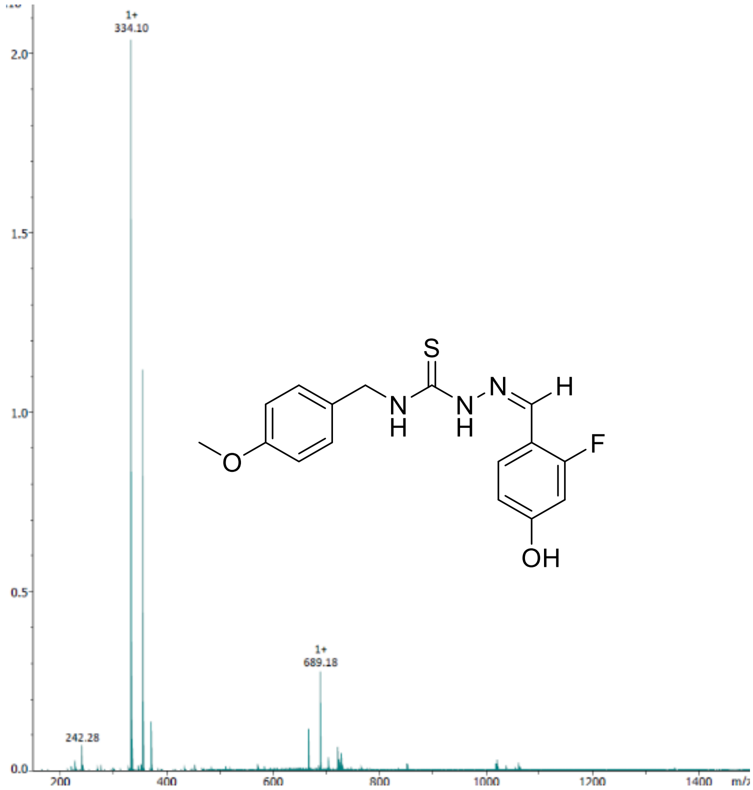

Figure S10: ESI Mass spectra of ligand HL^2

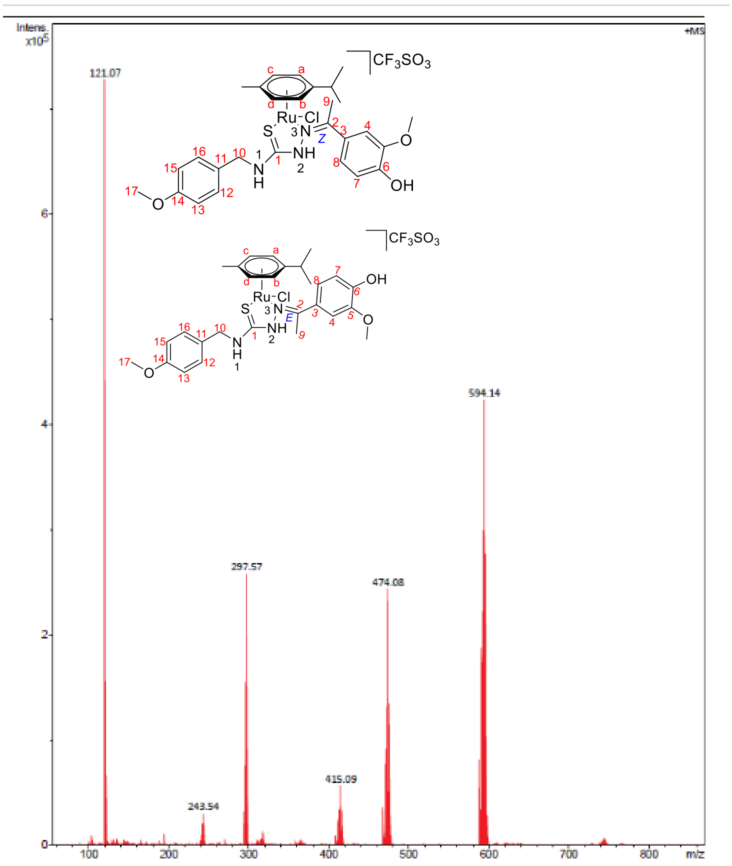

Figure S11: ESI Mass spectra of complex 1(TfO)

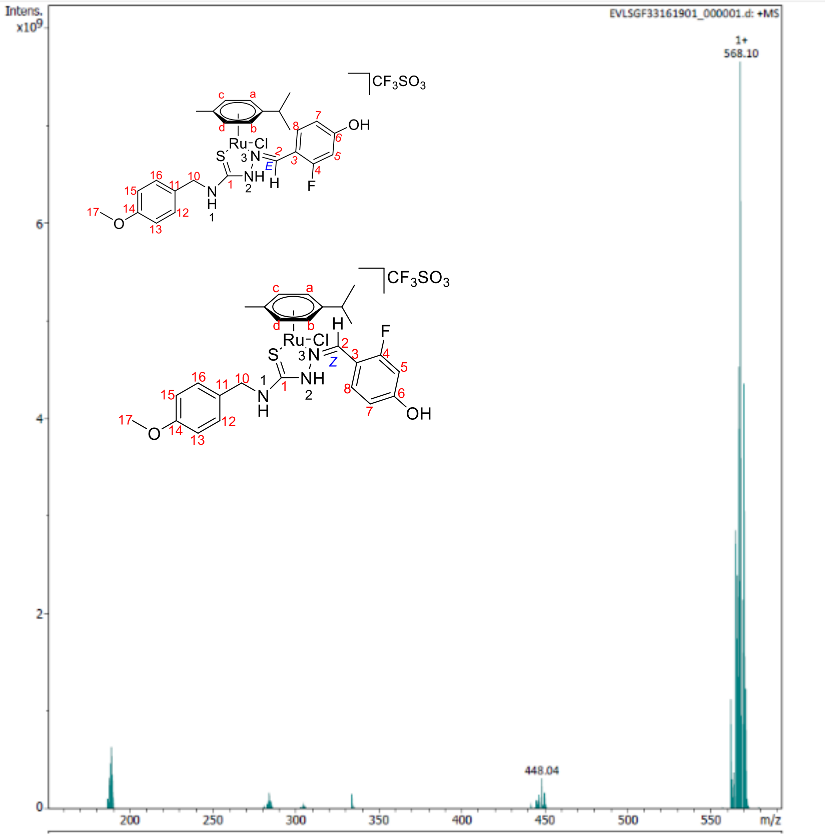

Figure S12: ESI Mass spectra of complex 2(TfO)

Infrared spectra

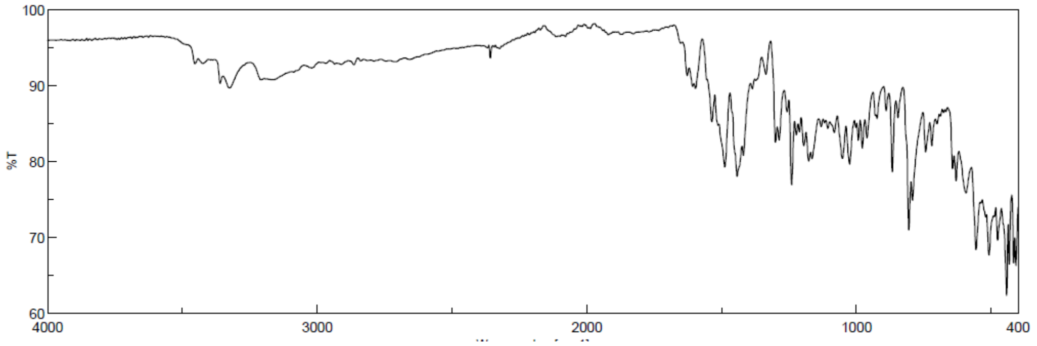

Figure S13: IR spectrum of ligand HL[1]

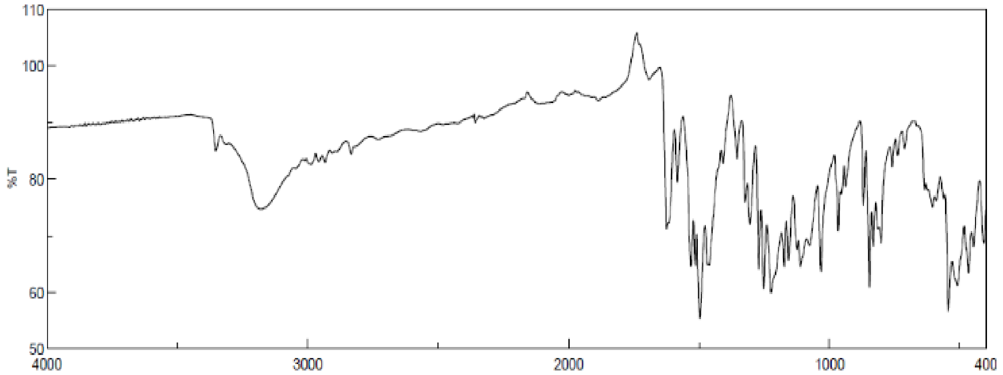

Figure S14: IR spectrum of ligand HL[2]

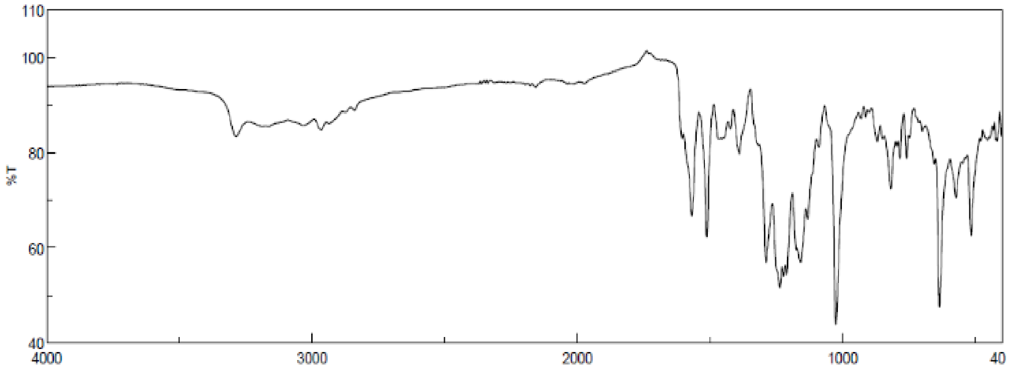

Figure S15: IR spectrum of complex 1(TfO)

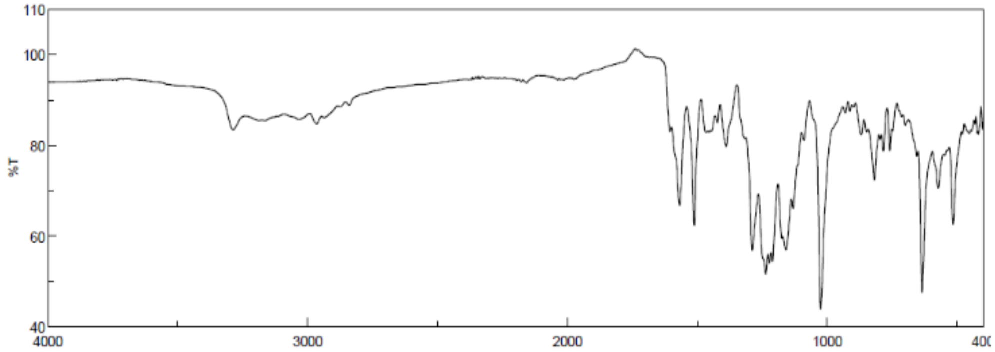

Figure S16: IR spectrum of complex 2(TfO)

Cyclic voltammetry

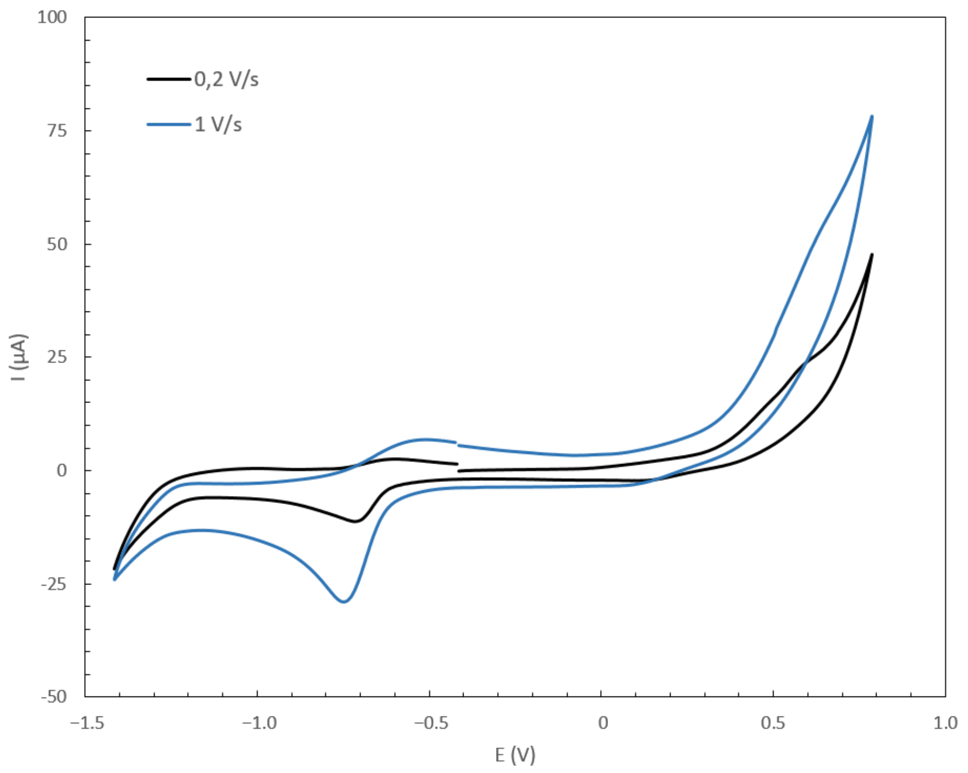

Figure S17: Cyclic voltammetry (CV) and square wave voltammetry (SWV) data of complex 1(TfO)

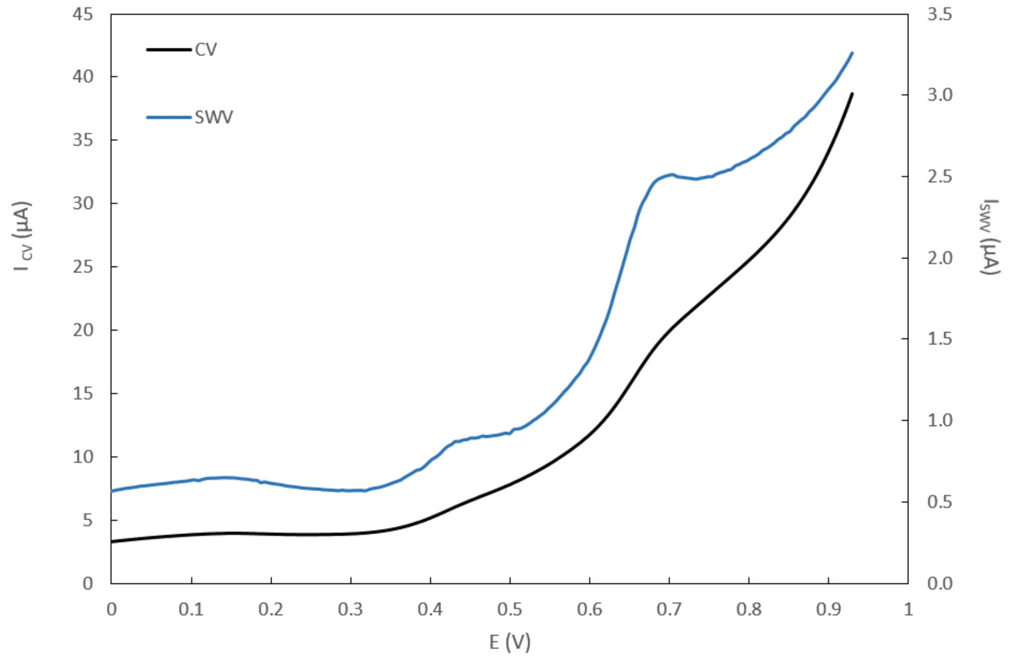

Figure S18: Cyclic voltammetry (CV) and square wave voltammetry (SWV) data of complex 2(TfO)

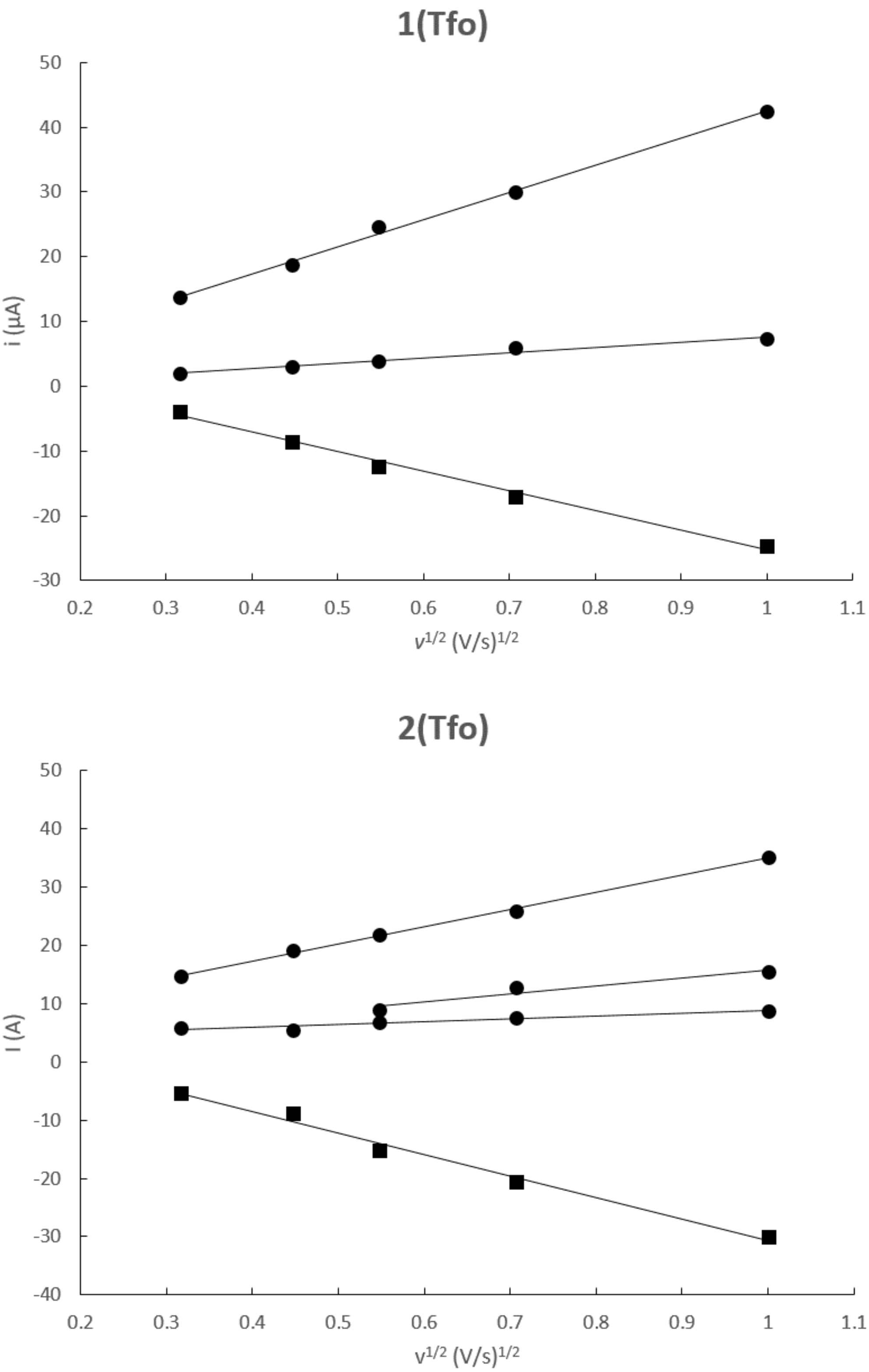

Figure S19: X: I_{pa} (●) and I_{pc} (▪) of redox waves for complexes 1(TfO) (top) and 2(TfO) (bottom) vs square root of scan rate.
